# Pan-cancer analysis of CDKN2A alterations identifies a subset of gastric cancer with a cold tumor immune microenvironment

**DOI:** 10.1186/s40246-024-00615-7

**Published:** 2024-05-31

**Authors:** Chao Deng, Zi-xi Li, Chen-jun Xie, Qing-lin Zhang, Ben-shun Hu, Mei-dan Wang, Jie Mei, Chen Yang, Zhangfeng Zhong, Ke-wei Wang

**Affiliations:** 1 Institute of Integrated Traditional Chinese and Western Medicine, Affiliated Hospital of Jiangnan University, No. 1000, Hefeng Rd, Wuxi, Jiangsu Province 214122 China; 2 Wuxi School of Medicine, Jiangnan University, Wuxi, China; 3 Departments of Gastroenterology, the Affiliated Wuxi People’s Hospital of Nanjing Medical University, Wuxi, China; 4 Department of Hepatobiliary Surgery, Affiliated Hospital of Jiangnan University, Wuxi, China; 5 Department of Oncology, The Affiliated Wuxi People’s Hospital of Nanjing Medical University, Wuxi, China; 6 Macao Centre for Research and Development in Chinese Medicine, Institute of Chinese Medical Sciences, University of Macau, Macau, Macao SAR 999078 China

**Keywords:** CDKN2A, Immunotherapy, Immune checkpoint inhibitor, Pan-cancer

## Abstract

**Background:**

Although CDKN2A alteration has been explored as a favorable factor for tumorigenesis in pan-cancers, the association between CDKN2A point mutation (MUT) and intragenic deletion (DEL) and response to immune checkpoint inhibitors (ICIs) is still disputed. This study aims to determine the associations of CDKN2A MUT and DEL with overall survival (OS) and response to immune checkpoint inhibitors treatment (ICIs) among pan-cancers and the clinical features of CDKN2A-altered gastric cancer.

**Methods:**

This study included 45,000 tumor patients that underwent tumor sequencing across 33 cancer types from four cohorts, the MSK-MetTropism, MSK-IMPACT, OrigiMed2020 and TCGA cohorts. Clinical outcomes and genomic factors associated with response to ICIs, including tumor mutational burden, copy number alteration, neoantigen load, microsatellite instability, tumor immune microenvironment and immune-related gene signatures, were collected in pan-cancer. Clinicopathologic features and outcomes were assessed in gastric cancer. Patients were grouped based on the presence of CDKN2A wild type (WT), CDKN2A MUT, CDKN2A DEL and CDKN2A other alteration (ALT).

**Results:**

Our research showed that CDKN2A-MUT patients had shorter survival times than CDKN2A-WT patients in the MSK MetTropism and TCGA cohorts, but longer OS in the MSK-IMPACT cohort with ICIs treatment, particularly in patients having metastatic disease. Similar results were observed among pan-cancer patients with CDKN2A DEL and other ALT. Notably, CDKN2A ALT frequency was positively related to tumor-specific objective response rates to ICIs in MSK MetTropism and OrigiMed 2020. Additionally, individuals with esophageal carcinoma or stomach adenocarcinoma who had CDKN2A MUT had poorer OS than patients from the MSK-IMPACT group, but not those with adenocarcinoma. We also found reduced levels of activated NK cells, T cells CD8 and M2 macrophages in tumor tissue from CDKN2A-MUT or DEL pan-cancer patients compared to CDKN2A-WT patients in TCGA cohort. Gastric cancer scRNA-seq data also showed that CDKN2A-ALT cancer contained less CD8 T cells but more exhausted T cells than CDKN2A-WT cancer. A crucial finding of the pathway analysis was the inhibition of three immune-related pathways in the CDKN2A ALT gastric cancer patients, including the interferon alpha response, inflammatory response, and interferon gamma response.

**Conclusions:**

This study illustrates the CDKN2A MUT and DEL were associated with a poor outcome across cancers. CDKN2A ALT, on the other hand, have the potential to be used as a biomarker for choosing patients for ICI treatment, notably in esophageal carcinoma and stomach adenocarcinoma.

**Supplementary Information:**

The online version contains supplementary material available at 10.1186/s40246-024-00615-7.

## Introduction

Immunotherapy represented by immune checkpoint inhibitors (ICIs) such as cytotoxic T-lymphocyte antigen-4 (CTLA-4), programmed cell death 1 (PD-1), and programmed death-ligand 1 (PD-L1) inhibitors can provide long-term clinical benefits for patients with various types of tumors [1]. Therefore, accurately identifying cancer subtypes sensitive to immunotherapy is a focus for cancer patients with non-surgical treatment.

Cyclin-dependent kinase inhibitor 2 A/2B (CDKN2A/2B) are both tumor suppressor genes with the second most frequent genetic alterations following p53. The human CDKN2A/B locus at 9p21 contains genes encoding p16INK4A, p14ARF and p15INK4B, which can inhibit CDK kinase activity and regulating G1 cell cycle. The inactivation of CDKN2A/2B can result in uncontrolled cell growth and proliferation [2]. Notably, deletion, mutation or hypermethylation of CDKN2A/2B are frequently observed in head and neck cancer, non-small cell lung cancer (NSCLC), prostate adenocarcinoma (PRAD), glioma, esophageal carcinoma, bladder cancer and T-cell lymphoma [2–8].

Currently, the association between CDKN2A alteration (ALT) and immunotherapy response is controversial. For example, Hildur Helgadottir et al. find a positive association between CDKN2A point mutation (MUT) and melanoma immunotherapy efficacy [9]. In a large cohort of 149 patients with stage III/IV NSCLC who receive radiotherapy, CDKN2A ALT predicts immunotherapy resistance [10]. In addition, for those individuals with advanced kidney renal clear cell carcinoma (KIRC), CDKN2A deletion is significantly associated with worse overall survival (OS) in patients with tumor-infiltrating T cells after PD-1 blockade [11]. In ‘cold’ tumors, patients with 9p21 deletion show low tumor-infiltrating white blood cells (TILs) and low objective response rates (ORRs) to ICIs [12]. The association of CDKN2A ALT with reduced benefit from ICI therapy is found in urothelial carcinoma (UC), not in five other cancers: esophagogastric cancer, head and neck cancer, NSCLC, renal cell carcinomas, and melanoma [7]. More research is needed to completely understand the mechanisms underlying the link between CDKN2A mutations/deletions and the responsiveness to ICI. We hypothesize that CDKN2A ALT leads to enrichment of tumor mutation-specific neoantigens on the cell surface, which is beneficial to the recognition and attack of immune cells. On the other hand, CDKN2A ALT leads to uncontrolled cell growth and proliferation caused by abnormal activation of CDK2/4/6. Thus, pan-cancer analysis of CDKN2A ALT may provide new insights into varying prognostic associations involving CDKN2A ALTs among cancer types or show some rationales for different levels of clinical response to ICIs.

In this study, we used more than 47,000 patients from the Memorial Sloan Kettering-Metastatic Events and Tropisms (MSK-MetTropism) [13], the Memorial Sloan Kettering-Integrated Mutational Profiling of Actionable Cancer Targets (MSK-IMPACT) [14], the China pan-cancer (OrigiMed2020) [15]and the Cancer Genome Atlas (TCGA) cohorts [16] to explore the characteristics of CDKN2A ALT, MUT or DEL and their associations with clinical outcomes and response to ICIs among pan-cancer patients treated or not treated with immunotherapy.

## Methods

### Patients and data collection

**Fig. 1:**
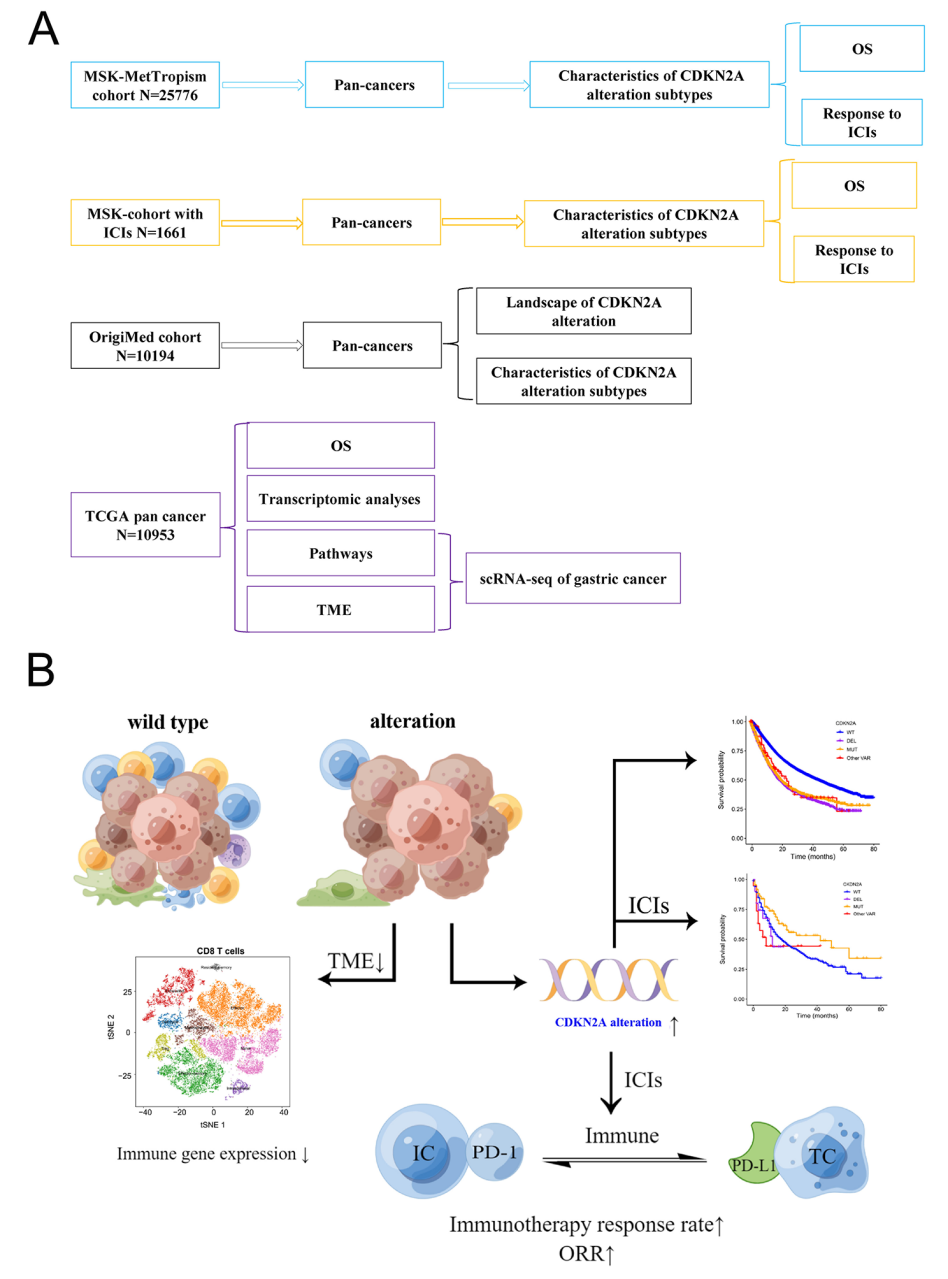
Overall schematic flow-chart of this study (**A**) and potential effects of CDKN2A alterations on the immune microenvironment and immunotherapy (**B**). MSK-MetTropism, Memorial Sloan Kettering-Integrated Mutation Profiling of Actionable Cancer Targets; MSK-IMPACT, Memorial Sloan Kettering-Integrated Mutational Profiling of Actionable Cancer Targets; ICI, immune checkpoint inhibitors; OS, overall survival; TCGA, the Cancer Genome Atlas

As the discovery cohort, we used a larger clinical-genomic database (*n* = 25,755) across 50 tumor types from the MSK-MetTropism cohort (Fig. 1A). In addition to somatic mutation data, demographic characteristics and clinical data were downloaded from the cBioPortal (https://www.cbioportal.org). Altogether, 24,503 patients were included after filtered missing CDKN2A somatic mutation information, survival, gender, age, MSI and duplicate samples (Table S1). In order to unravel the potential effects of different CDKN2A-mutant types on ICI, the CDKN2A-mutant sets should be divided into point mutation and deletion. The CDKN2A-ALT is an acronym for CDKN2A alterations, which include missense mutations, silent mutations, truncating mutations, inframe mutations, splice mutations, fusion mutations, amplifications, deep deletions, and structural variants. Three different forms of CDKN2A point mutations (CDKN2A MUT) exist: nonsense mutations, missense mutations, and silent mutations. Based on the cohorts provided by cBioPortal, only cases with CDKN2A homozygous deletion were available, so we defined CDKN2A homozygously deleted as CDKN2A DEL [17]. In all, in this study CDKN2A ALT subtypes included CDKN2A-MUT, CDKN2A-DEL, CDKN2A other-ALT and CDKN2A-WT groups.

The MSK-MetTropism cohort underwent sequencing at Memorial Sloan Kettering Cancer Center between November 18, 2013, and August 18, 2021, for a period of 1.6 years. These cases were part of the AACR Project Genomics Evidence Neoplasia Information Exchange (GENIE) 9.0-public database, which was a collaborative endeavor known as the AACR Project GENIE Consortium (AACR Project GENIE Consortium, 2017) [18]. The tumors were profiled utilizing the Memorial Sloan Kettering Integrated Molecular Profiling of Actionable Cancer Targets (MSK-IMPACT) clinical sequencing test. This technique makes use of a next-generation sequencing platform based on hybridization capture, according to description by Cheng et al. [19]. The MSK-IMPACT panel used for profiling was made up of multiple generations that examine a varied number of genes. These include 341 genes (*n* = 1,801 samples), 410 genes (*n* = 6,372 samples), and 468 genes (*n* = 17,602 samples). The panel detects somatic mutations, rearrangements, copy-number changes, TMB, chromosomal instability, and microsatellite instability in cancers. To provide a thorough picture of our study, we incorporated all patients and their genetic data from the discovery cohort, which was taken from the MSK-MetTropism cohort. It is important to emphasize that the patients in the MSK-MetTropism cohort received no ICIs medications. We used survival and genetic data to examine the frequency distribution of CDKN2A-ALT and its prognosis in pan-cancer patients.

### MSK-IMPACT cohort with ICIs

Next, we recruited 1661 patients from the MSK-influence cohort who received ICIs treatment to assess the influence of CDKN2A ALT on clinical outcomes in various cancer types after ICIs (https://www.cbioportal.org) [14]. The FDA-approved MSK-IMPACT assay is a next-generation sequencing-based diagnostic utilized at Memorial Sloan Kettering Cancer Centre for genetic profiling in precision oncology [20]. It discovered somatic mutations by examining certain gene panels. The impact of CDKN2A ALT, as evaluated by the MSK-IMPACT test, on overall survival in patients receiving ICI treatment was our primary focus. Patients in the cohort had a variety of cancers, including NSCLC, melanoma, renal cell carcinoma (RCC), bladder cancer, and head and neck squamous cell carcinoma. 146 patients in the cohort received anti-CTLA4 therapy, 1447 received anti-PD1 or PD-L1 therapy, and 189 received both types of immunotherapies.

### TCGA cohort

We utilized the TCGA cohort, encompassing a diverse array of cancer types, to examine how CDKN2A ALT affects patient prognosis in the absence of ICIs treatment. Analyzing the TCGA cohort, potentially as a validation cohort, would yield valuable insights into the influence of CDKN2A ALT on patient prognoses without ICI treatment, thereby enhancing our understanding in this field. The last cohort consisted of 11,160 patients across 33 tumor types from TCGA cohort. The CDKN2A ALT data was retrieved from the cBioPortal and the survival data including OS, progression-free survival (PFS), disease-free survival (DSS) and disease-free interval (DFI) was retrieved from TCGA pan-cancer clinical data resource (TCGA-CDR) [16]. TCGA pan-cancer RNA‐seq data were downloaded from UCSC Xena data portal (https://xenabrowser.net) for immune microenvironment assessment, immune function genes expression analysis, and pathway enrichment analysis. Figure 1B shows the flow chart of this study.

### OrigiMed 2020

We further used the big pan-cancer cohort from China (OrigiMed 2022) to undertake a comprehensive examination of the prevalence of CDKN2A ALT in the Asian population and its potential association with immunotherapy response. The next cohort comprised 10,194 patients (China Pan-cancer, OrigiMed 2020) across 25 tumor types from the Chinese population. The CDKN2A ALT data was retrieved from the cBioPortal (https://www.cbioportal.org). Additionally, TMB, MSI status and CNA data came from a previous study [21].

### Study outcomes

The primary outcomes were CDKN2A ALT frequency, survival status, immunotherapy regimen and response. We calculated the tumor type-specific and the total CDKN2A ALT frequency in each cohort. The immunotherapy involved in this study referred to ICIs therapy including CTLA-4 blockade, PD-1/PD-L1 blockade and combination. Immunotherapy response was evaluated using the objective response rate (ORR). Individuals who had a total or partial response to ICIs were classified as responders, denoting that the disease had undergone significant improvement (partial response) or complete eradication (complete response). Conversely, individuals who exhibited no improvement or experienced a consistent state of their condition were categorized as non-responders, characterized by either a stable or advancing sickness [22]. Tumor type-specific ORR mainly referred to the original publications [23–28]. The prediction of neoantigen burden involves gene sequencing and analysis of tumor tissue [29]. DNA sequences from the tumor tissue are analyzed to identify tumor-associated antigens with mutations, and bioinformatics algorithms are then used to predict the probability of their presentation on HLA molecules. For each sample, the fraction of genome changed (FGA) was calculated by taking the percentage of the genome with absolute log2 copy ratios greater than 0.2. The log2 copy-number ratios were calculated using the previously described approach [19]. This method allowed us to estimate the degree of genetic modifications in each sample by focusing on mutations that surpassed a specified log2 copy ratio threshold.

### TME and pathway enrichment analysis

To explore the effect of CDKN2A ALT on tumor microenvironment, we evaluated the infiltration levels of 22 diverse immune cells using the CIBERSORT algorithm in TCGA cohort [30]. Next, we investigated the association between CDKN2A ALT subtypes and canonical immune-functional genes, including immunoinhibitory molecules, immunostimulators, major histocompatibility complex (MHC) molecules, chemokines, and chemokine receptors in TCGA cohort [27, 31].

RNAseq data of eight tumor types: esophageal carcinoma (ESCA), kidney renal clear cell carcinoma (KIRC), kidney renal papillary cell carcinoma (KIRP), lung adenocarcinoma (LUAD), mesothelioma (MESO), pancreatic adenocarcinoma (PAAD), sarcoma (SARC), and stomach adenocarcinoma (STAD) with the most significant effect of CDKN2A ALT on OS were extracted. They were merged into a bigger cohort, among which differential gene expressions (DGEs) between CDKN2A ALT subtypes and CDKN2A-WT patients were obtained by R package DESeq2 [32]. Moreover, R package Cluster-Profiler was also used for gene set enrichment analysis (GSEA) [33].

### Single-cell RNA-sequencing data analysis

We conducted an analysis of single-cell RNA-sequencing data of gastric cancer. The data was downloaded from the GEO database (GSE206785) [34] (http://www.ncbi.nlm.nih.gov/geo/). We performed Seurat-based analysis to preprocess the single-cell data. Quality control measures, including the threshold for the number of detected genes per cell and the percentage of mitochondrial genes expressed, were similar to those used in Boxi Kang et al.’s study [34]. We identified markers for each main cell cluster using the FindAllMarker function. Cell type markers were sourced from the CellMarker website [35] and previous studies [34, 36–42].

### Cell culture, lentivirus transfection, and Western blot analysis

The human gastric cancer cell lines AGS and HGC-27 were obtained from the Chinese Academy of Sciences in Shanghai, China. The cells were cultured in RPMI1640 media containing 10% foetal bovine serum, 100 U/mL penicillin and streptomycin with 37 °C in a humid atmosphere containing 5% CO_2_. When transfecting the CDKN2A interference plasmid, transfection reagents and Opti-MEM may be applied, and the reagents were introduced into the cells for 8 h of culture before changing the culture media. The transfection efficiency should be assessed 24 h after transfection, and the culture medium should be replaced again for 24 h after successful transfection. The lentiviral particles encoding the CDKN2A shRNA were constructed by Hanbio, shanghai, China.

After full breaking of the lysate on ice for 1 h, cells were collected, cell lysate was added, and cell protein was extracted by centrifugation at low temperature. The protein content was determined using a BCA kit. The cell protein was transported through SDSPAGE and a polyvinylidene fluoride (PVDF) membrane after the sample buffer was deformed at high temperatures. Skim milk was sealed for 2 h, washed with TBST, and incubated with primary antibody overnight at 4 °C. After cleaning with TBST, the second antibody was incubated for 1 h, and the findings were obtained using ECL. The following primary antibodies were used for Western blotting: anti-CDKN2A/p14ARF (ab185620; Abcam), anti-CDKN2A/p16INK4a (ab241543; Abcam).

### Statistical analysis

OS, PFS, DSS and DFI were compared between CDKN2A ALT subtypes and CDKN2A-WT patients using the log-rank test and Cox regression analyses. The correlation between tumor-specific ORRs and CDKN2A ALT frequency was analyzed using the Pearson or Spearman correlation analysis. Continuous variables were compared in CDKN2A ALT-subtypes and CDKN2A-WT patients by the One-way ANOVA or kruskal-Wallis test. The functions of genes were determined using gene set enrichment analysis (GSEA), and the Benjamini-Hochberg method was used to adjust the p-value. All significance tests were two-sided, and *p* < 0.05 was considered statistically significant. Data were analyzed with R. version 4.1.1 (R Foundation for Statistical Computing).

## Results

### Association of alteration (ALT) with outcome measures in all tumors not treated with ICIs in the MSK-MetTropism cohort

**Fig. 2:**
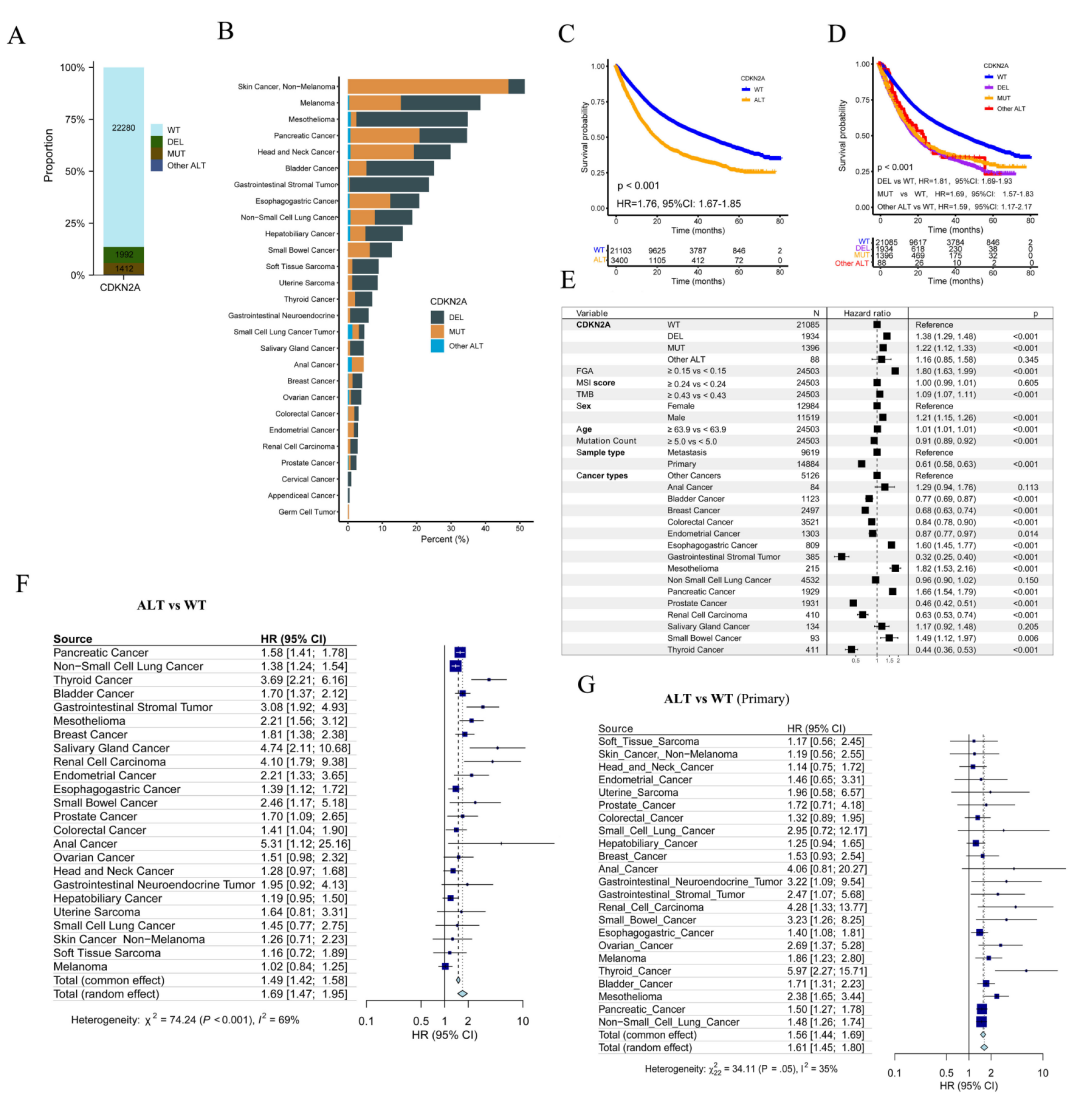
CDKN2A alterations in the MSK-MetTropism cohort. (**A**) The distribution of CDKN2A-DEL, CDKN2A-MUT and CDKN2A-WT patients. CDKN2A-MUT: CDKN2A point mutation, CDKN2A-DEL: CDKN2A deletion, CDKN2A-WT: CDKN2A-wide type. (**B**) Frequency of CDKN2A DEL, CDKN2A MUT and CDKN2A other ALT in each cancer type. (**C**) Over survival (OS) in CDKN2A-WT and CDKN2A-alteration (ALT) patients. (**D**) OS in prespecified subgroups including CDKN2A-DEL, CDKN2A-MUT, CDKN2A-WT and CDKN2A other-ALT groups. HR: hazard ratio, CI: confidence interval. (**E**) Multivariable Cox regression analysis presented the association between CDKN2A DEL, CDKN2A MUT, CDKN2A other ALT, various types of cancer and OS after controlling for other factors including FGA, MSI score, TMB, age, and metastasis status. The median values for FGA, MSI Score, TMB, Age, and Mutation Count are 0.15, 0.24, 4.32, 63.9, and 5.0, respectively. Patients with values below the median were assigned to the reference group, while those equal to or above the median were assigned to the treatment group. Forest plot of association between CDKN2A ALT and OS of all patient (**F**) and patients diagnosed with primary cancer (**G**)

Of 25,775 patients enrolled in the MSK-MetTropism cohort, 1992 (7.7%) were CDKN2A-DEL, 1412 (5.5%) were CDKN2A-MUT and 22,280 (86.4%) were CDKN2A-WT (Fig. 2A). This cohort included 27 cancer types, and Skin Cancer (Non − Melanoma) had the highest CDKN2A ALT frequency (46.7% were CDKN2A-DEL and 4.8% were CDKN2A-MUT), followed by melanoma, mesothelioma, pancreatic cancer, head and neck cancer, bladder cancer and gastrointestinal stromal tumor (Fig. 2B). Of 24,503 patients with complete OS data, CDKN2A-DEL patients had the most FGA, MSI score and largest number of patients having metastatic disease, while those CDKN2A-DELMUT patients had the biggest TMB and mutation count (Table S1). Log-rank test showed that CDKN2A-ALT (hazard ratio [HR], 1.76; 95% CI, 1.67–1.85; Fig. 2C), CDKN2A-DEL (HR, 1.81; 95% CI, 1.69–1.93; Fig. 2D), CDKN2A-MUT (HR, 1.69; 95% CI, 1.57–1.83; Fig. 2D) patients had poorer OS than CDKN2A-WT patients. Moreover, after adjusting FGA, MSI, TMB, age, metastasis status and gender using multivariable Cox regression, CDKN2A-DEL, CDKN2A-MUT, CDKN2A-Other ALT still could predict OS of participants (HR, 1.38; 95% CI, 1.29–1.48; 1.22; 95% CI, 1.12–1.33; 1.16; 95% CI, 0.85–1.58; Fig. 2E).

Subsequently, a pan-cancer survival analysis uncovered a consistent association between CDKN2A ALT and inferior OS in various cancer types (Fig. 2F). Likewise, a comparable association was observed in patients with CDKN2A-DEL (Figure S1E). However, this particular observation was limited to patients diagnosed with six specific types of cancer and having CDKN2A-MUT (Figure S1B). Significantly, a meta-analysis confirmed the positive correlation between CDKN2A ALT, CDKN2A MUT, CDKN2A DEL, and unfavorable prognosis in pan-cancers (Fig. 2F, Figure S1B and E).

Patients with CDKN2A ALT had poor prognosis in as many as 12 primary cancer types (Fig. 2G). Furthermore, when it came to metastatic cancers, we discovered that patients with CDKN2A ALT had even worse prognostic outcomes in 10 different cancer categories (Figure S1A). Additionally, our study enabled us to identify a specific subset of cancers that consistently displayed these distinctive prognostic characteristics. Examples of such cancers included pancreatic cancer, bladder cancer, thyroid cancer, renal cell carcinoma, and gastrointestinal stromal tumor (Fig. 2G and Figure S1A). Patients with CDKN2A DEL had similar outcomes (Figure S1F and G). CDKN2A MU were associated with poor prognosis in as many as six primary tumor types (Figure S1C). Furthermore, when it came to metastatic cancers, patients with CDKN2A MU had even worse prognostic outcomes in four different tumor categories (Figure S1D). Additionally, stratified survival analysis suggested that CDKN2A-ALT patients were shorter OS than CDKN2A-WT patients across multiple levels of CNA, MSI, TMB, age, mutation count, gender, tumor purity and race (Figure S1H). To determine the association between CDKN2A ALT and response to ICIs among pan-cancer patients treated with immunotherapy (CTLA-4 blockade, PD-1/PD-L1 blockade or combination), tumor-specific ORRs were summarized according to some cancer immunotherapy studies. Of note, with the frequency of CDKN2A ALT extracted from the MSK-MetTropism cohort, we found a significant correlation between CDKN2A ALT frequency and ORRs (*r*, 0.58; *P* = 0.002; Figure S1I), suggesting that cancer patients with CDKN2A ALT might benefit more from immunotherapy.

### Association of CDKN2A ALT with outcome measures in all tumors treated with ICIs in the MSK-IMPACT cohort

**Fig. 3:**
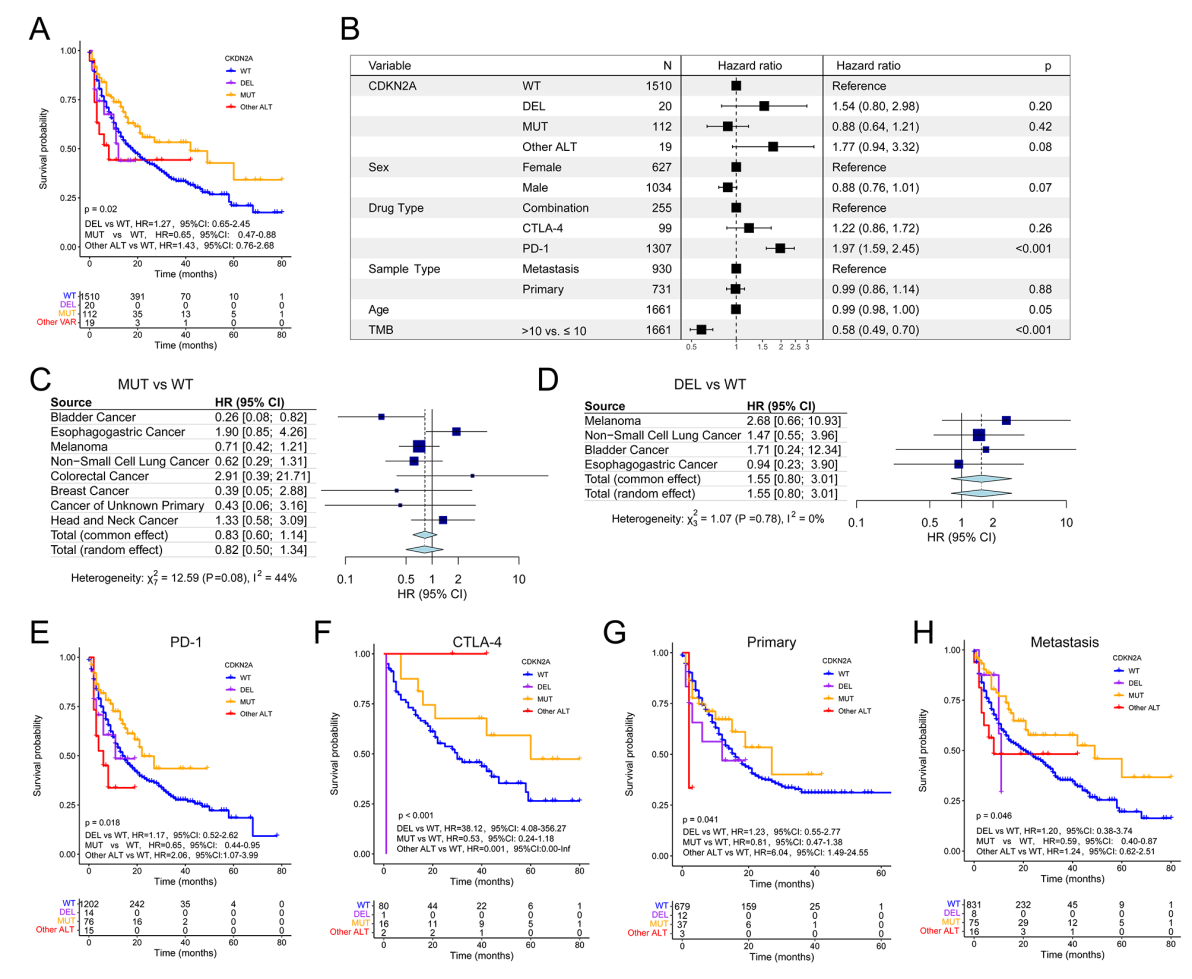
Associations of CDKN2A alterations with OS and objective response rates to ICIs in the MSK-IMPACT cohort. (**A**) OS in prespecified subgroups including CDKN2A-DEL, CDKN2A-MUT, CDKN2A-WT and other ALT groups. **(B**) Multivariable Cox regression analysis presented the association between CDKN2A-DEL, CDKN2A-MUT and CDKN2A-other ALT and OS after controlling for other factors including drug type, sex, TMB, age, and metastasis status. Forest plot of association between CDKN2A-MUT (**C**) and CDKN2A-DEL (**D**) and OS. OS of CDKN2A-DEL, CDKN2A-MUT, CDKN2A-WT and other ALT groups with PD-1 (**E**) and CTLA-4 (**F**) inhibitors treatment, primary cancer (**G**) or metastasis (**H**)

We used 1661 patients from the MSK-IMPACT cohort treated with ICIs to assess changing effect of CDKN2A ALT on clinical outcomes of some cancer types after immunotherapy. Association of CDKN2A ALT with prolonged OS reached statistical significance in all patients (HR, 0.77; 95% CI, 0.59–0.99; Figure S2A). Particularly noteworthy, CDKN2A-MUT patients outlived CDKN2A-WT patients after immunotherapy (HR, 0.65; 95% CI, 0.47–0.88; Fig. 3A), whereas patients with CDKN2A DEL did not (HR, 1.27; 95% CI, 0.65–2.45; Fig. 3A). Moreover, multivariable Cox regression showed that CDKN2A DEL, CDKN2A MUT, CDKN2A other ALT could not predict the OS of participants after immunotherapy (HR, 1.54; 95% CI, 0.80–2.98; 0.88; 95% CI, 0.64–1.21; 1.77, 95% CI, 0.94–3.32; Fig. 3B). Next, in the MSK-IMPACT cohort with ICIs treatment, significant associations of CDKN2A MUT, DEL, and ALT with OS were not found in esophagogastric cancer, breast cancer, melanoma, and NSCLC except for bladder cancer (Figure S2B; Fig. 3C and D), in contrast to the MSK-MetTropism cohort (Fig. 2F, Figure S1B and E). These disparities may be due to immunotherapy used in the MSK-IMPACT cohort’s 1661 patients. Similarly, a random effects meta-analysis of studies found no significant association between CDKN2A MUT, DEL, ALT, and OS in pan-cancer patients (Fig. 3C and D, Figure S2B).

Furthermore, survival analysis stratified by immunotherapy types suggested that CDKN2A-MUT patients treated with PD-1/ PD-L1 inhibitors (HR, 0.65; 95% CI, 0.44–0.95; Fig. 3E) but not CTLA-4 inhibitors (HR, 0.53, 95%CI, 0.24–1.18; Fig. 3F) or a combination of the two (HR, 1.15, 95%CI, 0.56–2.40; Figure S2C) had a better OS than CDKN2A-WT patients. Next, CDKN2A DEL was not related to OS in either primary or metastasis cancers (Fig. 3G and H; HR, 1.23; 95% CI, 0.55–2.77; metastasis, HR, 1.20; 95% CI, 0.38–3.74). Conversely, CDKN2A-MUT patients exhibited longer OS than CDKN2A-WT patients with metastatic disease (HR, 0.59; 95% CI, 0.40–0.87; Fig. 3H). Patients with primary cancers did not show this difference (HR, 0.81; 95% CI, 0.47–1.38; Fig. 3G). We combined the five cancer types with the highest CDKN2A ALT frequency in the MSK-MetTropism cohort and the TCGA cohort. Three cancer types were eventually identified and survival analyses were performed, and it was found that CDKN2A-ALT had a worse OSin PAAD and MESO except HNSC (Figure S2D-F). Based on this, we found that CDKN2A-ALT has a poor prognostic effect in some tumors, so pan-cancer analysis was subsequently performed.

### **Associations of CDKN2A ALT with clinical outcomes in TCGA cohort**

**Fig. 4:**
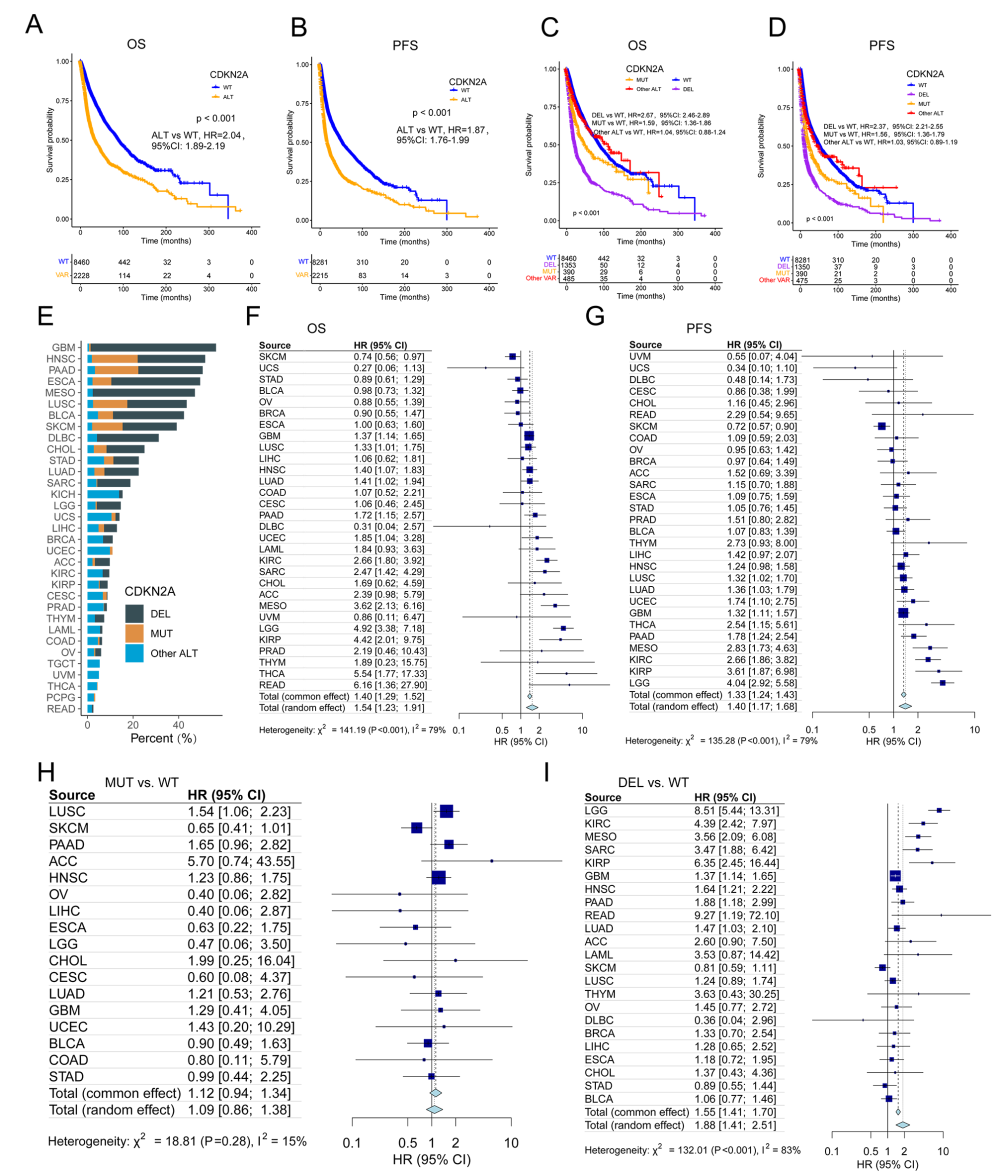
Associations of CDKN2A alterations with clinical outcomes in the TCGA cohort. OS (**A**) and PFS (**B**) in CDKN2A-WT and CDKN2A-ALT patients. OS (**C**) and PFS (**D**) in CDKN2A-DEL, CDKN2A-MUT, CDKN2A-WT and CDKN2A-other ALT patients. (**E**) Frequency of CDKN2A-DEL, CDKN2A-MUT and CDKN2A-other ALT in each cancer type. Forest plot of association between CDKN2A ALT and OS (**F**) and PFS (**G**). Forest plot of association between CDKN2A MUT(**H**) and DEL(**I**) and OS

The TCGA cohort (*n* = 10,953) was used to assess the effect of CDKN2A ALT on clinical outcomes. The CDKN2A ALT was associated with poorer OS (HR, 2.04; 95% CI, 1.89–2.19; Fig. 4A) and progression-free survival (PFS; HR, 1.87; 95% CI, 1.76–1.99; Fig. 4B). A pan-cancer survival analysis revealed that CDKN2A-ALT patients had a shorter OS and PFS than CDKN2A-WT patients in multiple cancer types, including LGG, KIRC, MESO, kidney renal papillary cell carcinoma (KIRP), pancreatic adenocarcinoma (PAAD), head and neck squamous cell carcinoma (HNSC), glioblastoma multiforme (GBM), lung adenocarcinoma (LUSC, Fig. 4F and G). In addition, 399 (3.6%) of all patients were CDKN2A-MUT, 1378 (12.6%) were CDKN2A-DEL, and 8673 (79.2%) were CDKN2A-WT (Figure S3A). In most tumor types, the frequency of CDKN2A-DEL was greater than that of CDKN2A MUT (Fig. 4E).

Next, all patients with CDKN2A MUT (OS, HR, 1.59, 95%CI: 1.36–1.86, Fig. 4C; PFS, 1.56, 95%CI: 1.36–1.79, Fig. 4D) or CDKN2A DEL (OS: HR, 2.67, 95%CI: 2.46–2.89, Fig. 4C; PFS, 2.37, 95%CI: 2.21–2.55, Fig. 4D) had a worse prognosis in TCGA cohort. Meta-analysis suggested that patients with CDKN2A DEL had poor OS (HR, 1.88, 95%CI: 1.41–2.51, Fig. 4I) and PFS (HR, 1.59, 95%CI: 1.26-2.00, Figure S3C), while those with CDKN2A MUT did not (OS: HR, 1.12, 95%CI: 0.94–1.34, Fig. 4H and PFS, 1.18, 95%CI: 0.95–1.46, Figure S3B). However, after extracting tumor-specific ORRs from some cancer immunotherapy studies, the correlation of CDKN2A ALT frequency with ORRs did not reach statistical significance (r, 0.2; *p* = 0.28) in TCGA cohort (Figure S3D). In conclusion, CDKN2A ALT was associated to a worse OS in patients who were not treated with ICIs; the addition of ICIs also might improve clinical outcomes in pan-cancer patients.

### **Associations of CDKN2A ALT with clinical outcomes in the merged cohort**

Considering that the MSK-IMPACT cohort was a cohort after ICIs treatment, while the OrigiMed 2022 cohort lacked survival outcomes, we only merged the MSK-MetTropism cohort and TCGA cohort to explore the impact of CDKN2A ALT on patient survival. In the merged cohort, we found that CDKN2A ALT was associated with poorer OS (HR, 1.95; 95% CI, 1.86–2.03; Figure S3E). A pan-cancer survival analysis revealed that CDKN2A-ALT patients had a shorter OS than CDKN2A-WT patients in 23 cancer types, including thymic epithelial tumor, thyroid cancer, renal non clear cell carcinoma, salivary gland cancer, gastrointestinal stromal tumor, adrenocortical carcinoma and so on. Among the 37 types of cancers in the merged cohorts, 23 cancer types with CDKN2A ALT exhibited shorter OS compared to CDKN2A-WT patients (Figure S3F). These cancer types include some of the most common malignancies such as breast cancer, glioma, pancreatic cancer, prostate cancer, head and neck cancer, non-small cell lung cancer, gastrointestinal stromal tumor, bladder cancer, colorectal cancer, and hepatobiliary cancer. Additionally, a meta-analysis indicated that patients with CDKN2A ALT had poor OS (HR, 1.89, 95%CI: 1.54–2.32, Figure S3F).

### **Landscape of CDKN2A ALT in a China pan-cancer cohort (OrigiMed 2022)**

**Fig. 5:**
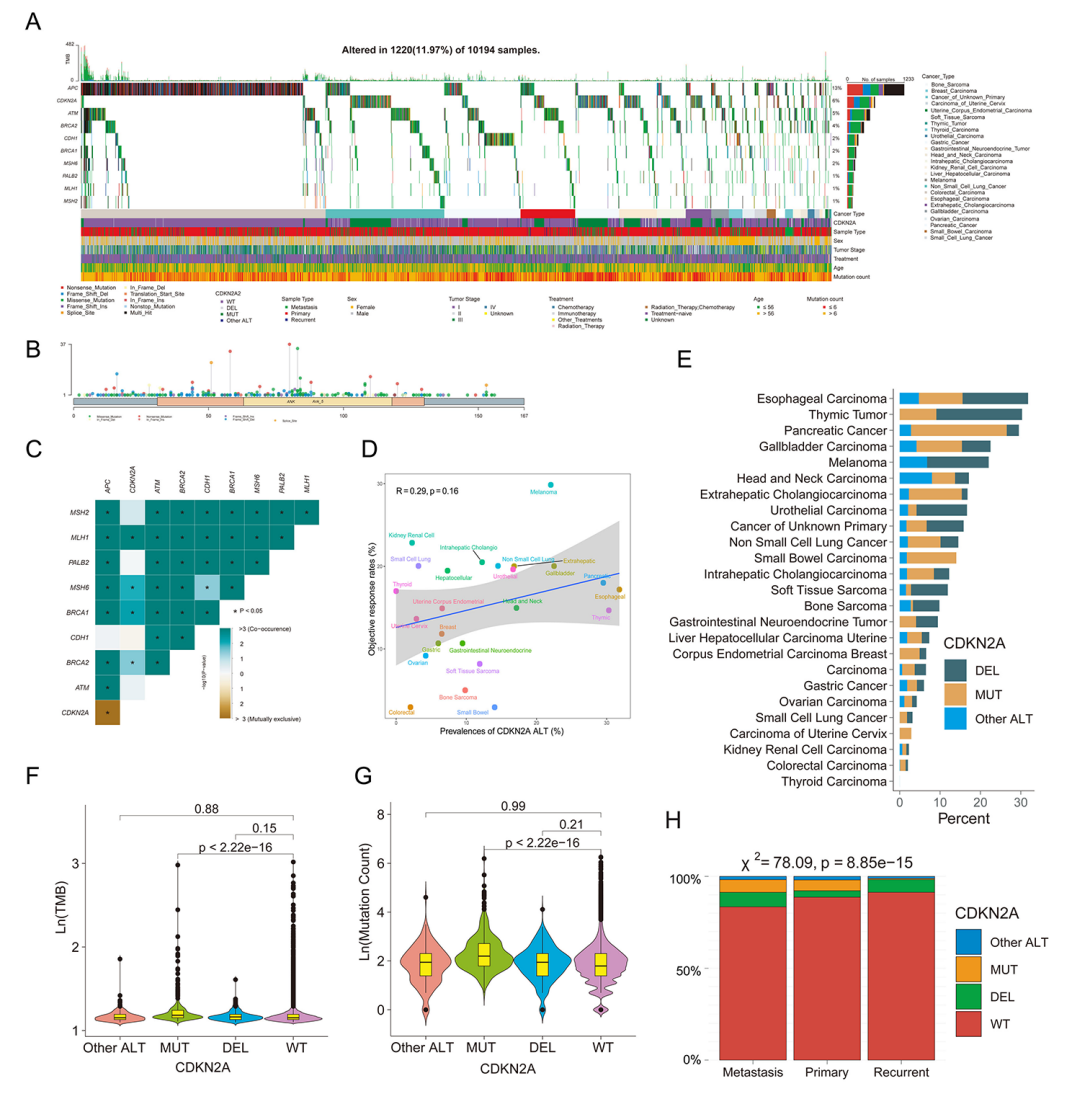
Clinical and CDKN2A alterations characteristics in the OrigiMed cohort. (**A**) Landscape of somatic CDKN2A alterations across 25 cancer types. (**B**) The alterations sites of CDKN2A in the OrigiMed cohort. (**C**) Heatmap showing the pattern of co-occurrence/mutual exclusivity of CDKN2A, APC, ATM, BRCA1, BRCA2, CDH1, MLH1, MSH2, MSH6, and PALB2. (**D**) Correlation between CDKN2A ALT frequency and objective response rates to ICIs. (**E**) Frequency of CDKN2A-DEL, CDKN2A-MUT and CDKN2A-other ALT in each cancer type. Comparison of TMB (**F**), mutation count (**G**) and metastasis (**H**) among four subgroups

We explored the prevalence of CDKN2A ALT among 10,194 patients across 25 cancer types in the OrigiMed 2022 cohort with clinical data (Fig. 5A). In total, 1220 patients (11.97%) had CDKN2A-ALT, 605 patients (5.93%) had CDKN2A-MUT, and 421 patients (4.13%) had CDKN2A-DEL (Figure S4A). Mutations diagram circles were colored with respect to the corresponding ALT types and showed that the H83Y was the most frequent somatic mutation site (Fig. 5B). Among nine genes germline pathogenic variants including APC, ATM, BRCA1, BRCA2, CDH1, MLH1, MSH2, MSH6, and PALB2 being associated with the risk of gastric cancer [43], CDKN2A had co-occurrence with MLH1, MSH6, BRCA1 and BRCA2 (Fig. 5C). To explore the immunotherapy response, we found the correlation between CDKN2A ALT frequency and ORRs in the OrigiMed 2022 cohort did not reach statistical significance (r, 0.29; *p* = 0.16; Fig. 5D). The highest frequency of CDKN2A ALT was observed in patients with esophagogastric cancer, followed by thymic tumor, pancreatic cancer, gallbladder carcinoma and melanoma (Fig. 5E). Patients with CDKN2A MUT had more elevated TMB, mutation count and age than those with CDKN2A WT (Fig. 5F, G and Figure S4B). Patients with CDKN2A ALT tended to have metastatic disease, be male and stage IV (Fig. 5H, and Figure S4C, D). Patients with CDKN2A MUT showed similar results. Those CDKN2A DEL patients were more likely to be recurrent and older than CDKN2A WT patients (Fig. 5H and Figure S4B). Furthermore, the treatment distribution differed significantly among four CDKN2A VAR subtypes (Fig. 4SE).

### TME in TCGA pan-cancer cohort

**Fig. 6:**
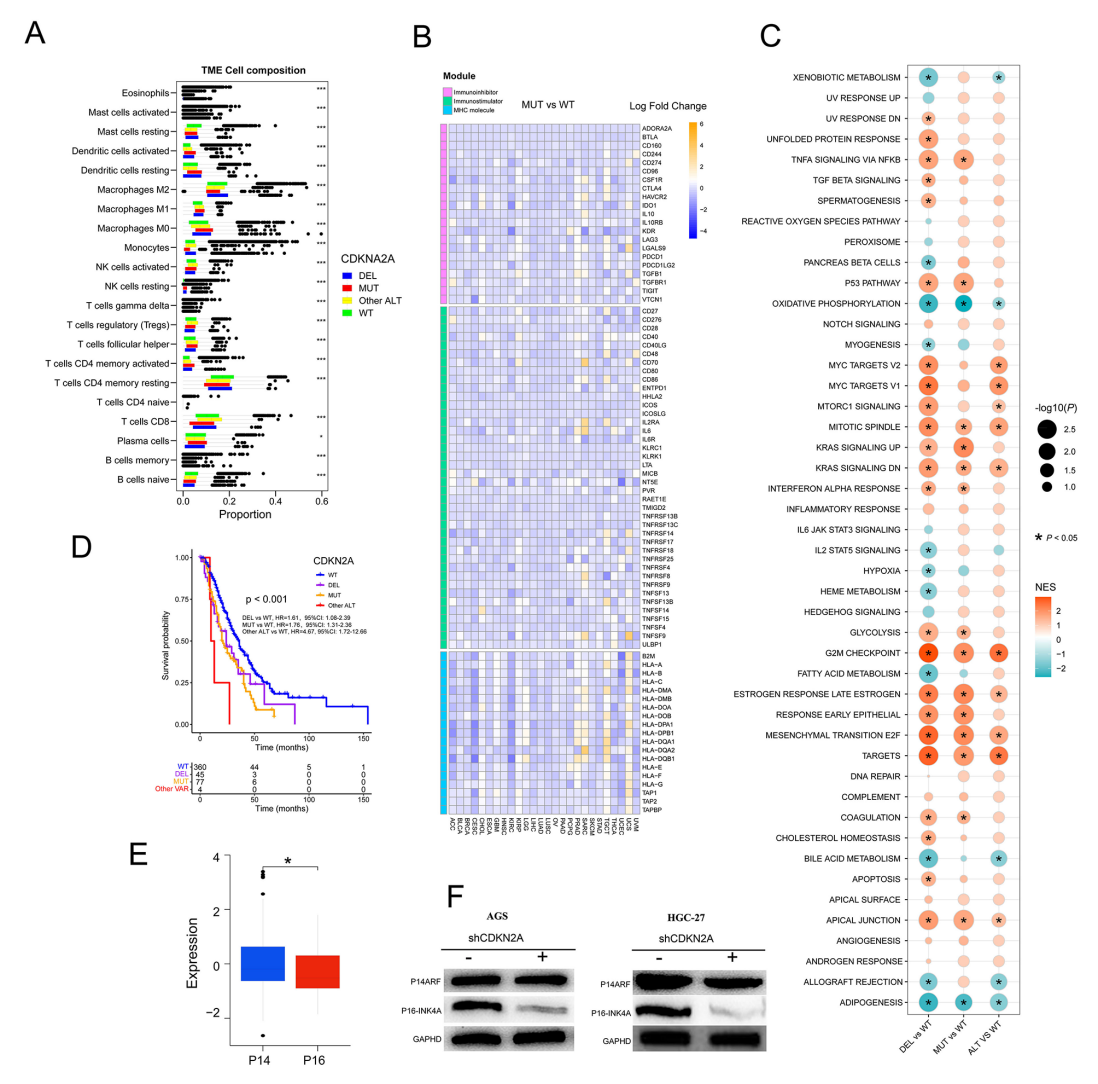
Assessment of immune infiltration, immune signatures, and pathway enrichment in CDKN2A-mutated pan-cancer patients in the TCGA cohort. Associations of CDKN2A ALT with immune cells infiltration (**A**), immune-related genes (**B**), signaling pathways (**C**) in TCGA Cohort (**D**) OS in CDKN2A-DEL, CDKN2A-MUT, CDKN2A-WT and CDKN2A-other ALT patients from Smita Sihag esophagogastric cancer cohort. (**E**) Protein comparison of p14 ARF (p14) and p16INK4a (p16) in CDKN2A-mutated STAD patients from the TCGA cohort. (**F**) Western blotting showed the expression of p16 and p14 in stomach cancer cell lines after CDKN2A knockdown

Pan-cancer immunogenicity can be influenced by genomic mutation. In the TCGA pan-cancer cohort, we used CIBERSORT to assess immune cell infiltration of tumor tissue. NK cells activated, monocytes, Tregs, B cells naive, T cells CD8, T cells CD4 memory resting, and macrophages M2 were lower in CDKN2A-MUT or DEL patients’ tumor tissue than in CDKN2A-WT patients, implying an immune cold tumor immune microenvironment in TCGA pan-cancer with CDKN2A VAR (Fig. 6A). Similarly, CDKN2A-MUT or DEL patients had lower expression of critical immune function genes in most tumor types, which could influence immune regulators in the tumor microenvironment, such as immunoinhibitory molecules, immune stimulators, MHC molecules, chemokines, and chemokine receptors, than CDKN2A-WT patients (Fig. 6B, Figure S5 A and B). These findings suggested that CDKN2A MUT or DEL could result in a cold tumor immune microenvironment and primary resistance to immune checkpoint therapy.

### Pathways associated with CDKN2A ALT in TCGA pan-cancer cohort

We evaluated at eight cancer types with high frequencies of CDKN2A VAR and a significant influence on patient outcomes: ESCA (Esophageal carcinoma), KIRC (Kidney renal clear cell carcinoma), KIRP (Kidney renal papillary cell carcinoma), LUAD (Lung adenocarcinoma), MESO (Mesothelioma), pancreatic (PAAD), SARC (Sarcoma) and Stomach adenocarcinoma (STAD). To further assess pathways associated with CDKN2A mutational status, GSEA were implemented on gene sets for all patients with and without CDKN2A ALT on in TCGA above eight cancer types-cohorts. Several pathways of tumorigenesis, development and metastasis, including TNFA signaling via NFKB, MYC targets, mTORC1 signaling, KRAS signaling, G2M checkpoint, Estrogen response, P53 pathway, EMT and E2F targets were upregulated in CDKN2A-MUT or DEL tumors (Fig. 6C). Of note, inflammatory response and IL6 JAK STAT3 signaling were not significantly altered among four groups, while IL2 STAT5 signaling were downregulated in CDKN2A-DEL tumors, and IFN ALPHA response were upregulated in CDKN2A-MUT and DEL tumors (Fig. 6C). Expectedly, hypoxia was significantly downregulated in CDKN2A-DEL tumors than in CDKN2A-WT tumors. Hypoxia can attenuate the function of cytotoxic T cells and attract regulatory T cells to reduce tumor immunogenicity [44].

Three pan-cancer cohorts (the MSK MetTropism, TCGA, and OrigiMed 2020 cohorts) found high CDKN2A ALT rates and significant survival effects in esophageal/stomach cancer, and Smita Sihag et al. found that CDKN2A had strong association with poor OS in 487 esophageal/stomach cancer patients with targeted sequencing [45]. Furthermore, utilizing the latter cohort, we found that all patients with CDKN2A MUT (OS: HR, 1.76, 95%CI: 1.31–2.36, Fig. 6D) or CDKN2A DEL (OS: HR, 1.61, 95%CI: 1.08–2.39, Fig. 6D) tended to have poor OS than those with WT. We used the TCGA gastric cancer dataset to compare the expression levels of the p14 ARF (p14) and p16INK4a (p16) proteins in the CDKN2A mutant group and found that the p16 protein was considerably less expressed than the p14 protein (Fig. 6E) [46]. Next, western blotting revealed a downregulation of p16 but no change in p14 expression in CDKN2A knockdown gastric cancer cell lines (AGS and HGC-27, Fig. 6F). Our findings demonstrated that CDKN2A VAR promoted cell proliferation by reducing expression of p16 with function as a regulator of the cell cycle of inhibiting CDK4 and CDK6 in gastric cancer.

### scRNA-seq of gastric cancer cells identifies cold immune microenvironment in CDKN2A ALT tissues

**Fig. 7:**
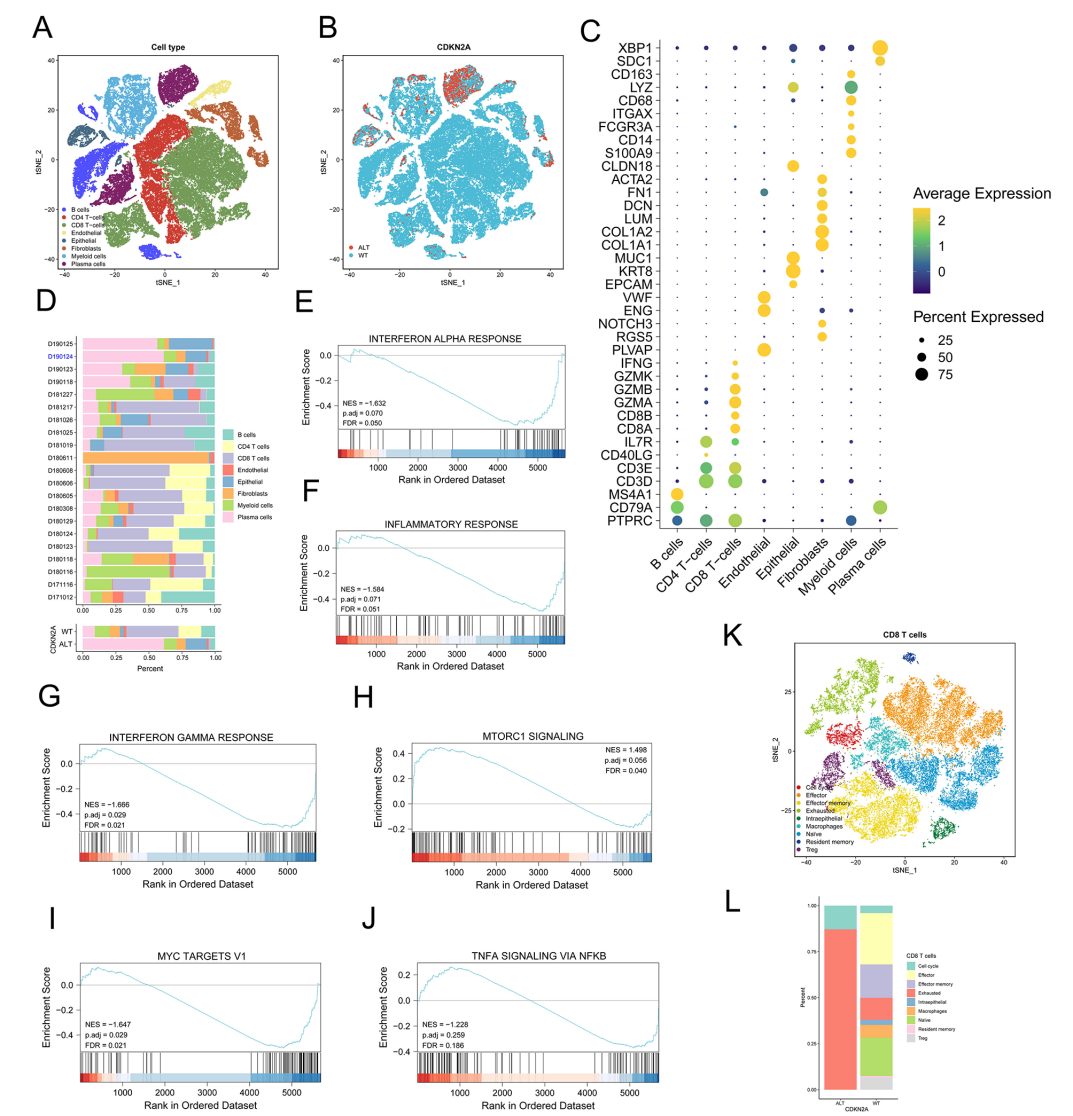
Single-cell transcriptional profiling of human GC. (**A**) scRNA-seq tSNE projection of 59,594 single cells from 21 primary GCs samples. (**B**) tSNE plot of 59,594 single cells from 21 primary GCs samples, color-coded by group. (**C**) Dot plot showing the gene expression patterns of cell-type marker genes in the scRNA-seq data. (**D**) Proportions of cell types in individual samples (above) and in different groups (bottom). scRNA-seq, single-cell RNA sequencing. (**E-J**) The GSEA plot shows enrichment of several pathways in CDKN2A ALT group. FDR < 0.05 is considered as significantly enriched. (**K**) tSNE plot of CD8 + T cells from 21 primary GCs samples. (**L**) The proportion of each CD8^+^ T cell cluster in CDKN2A-WT and CDKN2A- ALT patients

Previous studies in gastric cancer have reported that CDKN2A loss confers a cold tumor immune microenvironment [12]. We analyzed the scRNA-seq profiles of 21 primary gastric cancers (*n* = 59,594 cells), 20 of which were CDKN2A WT and one were CDKN2A-mutant (Missense Mutation) based on WES [34], to determine if CDKN2A ALT in gastric cancer was significantly associated with decreased inflammation. To shed light on cell populations present in gastric tumors, we clustered the 59,594 cells and identified 15 subclusters (Figure S6A) which were subsequently determined to be eight cell subgroups including B cells (MS4A1, CD79A), CD4 T − cells (CD3D, IL7R), CD8 T − cells (GZMA, GZMB, CD3E), endothelial cells (PLVAP, NOTCH3, ENG), epithelial cells (MUC1, KRT8), fibroblasts cells (COL1A1, COL1A2), myeloid cells (CD14, S100A9) and plasma cells (SDC1, XBP1) (Fig. 7A and C). A high proportion of CD8 T cells was observed in patients with CDKN2A WT (39.33%) while a low proportion of CD8 T cells was observed in patients with CDKN2A ALT (1.32%) (Fig. 7B and D). Those CDKN2A other ALT samples from TCGA cohort used in this study were shown to display more CD8 T cells (Fig. 6A). Similarly, three immune-related pathways including interferon alpha response, inflammatory response and interferon gamma response were inhibited in the CDKN2A ALT group (Fig. 7E-G). The mTOR signaling, MYC targets v1 signaling, and TNFA signaling via NFKB signaling were low enriched in the CDKN2A ALT group (Fig. 7H-J).

To understand the characteristics for the tumor cells, we performed re-clustering of CD8 T cells demonstrating nine sub-cell population including cell cycle cells (TUBA1B, HIST1H4C, KIAA0101, STMN1 and TUBB), effector cells (NKG7), effector memory cells (GZMB, ANXA1 and CREM), exhausted cells (CTLA4 and LAG3), intraepithelial cells (LIPF), macrophages cells (HSPA1A, HSPA1B and CXCL8), naïve cells (IL7R), resident memory cells (ANXA1 and GZMB) and Treg cells (IL2RA) (Fig. 7K and Figure S6B). The examination of the gastric cancer scRNA-seq data revealed that the tumors from CDKN2A-ALT patients had more exhausted CD8 T cells than those from CDKN2A WT patients (Fig. 7L). The results demonstrated the presence of a cold immune microenvironment in CDKN2A-ALT tumors, which may account for the resistance to ICIs. Our results highlighted the potential of scRNA-seq data to illuminate previously unknown characteristics of the immune microenvironment in cancers and promote the development of more effective immunotherapies.

## Discussion

In this study, we examined the prevalence of CDKN2A MUT and DEL in 25,755 tumor samples from 33 cancer types in the MSK-MetTropism cohort and discovered associations between CDKN2A MUT or DEL and clinical outcomes in pan-cancer patients with/without ICIs treatment. The associations were investigated further in three additional cohorts (*n* = 23,015). Patients with CDKN2A ALT who were not treated with ICIs had a shorter OS, while those with CDKN2A ALT who were treated with ICIs, especially those with metastatic disease, had a longer OS. The CDKN2A ALT was also positively correlated with ORRs to ICIs and with decreased anti-tumor immunity. This study used a larger sample of different teams from different backgrounds to ensure that the results of the study could be generalized and to highlight the potential value of CDKN2A ALT as a predictive biomarker of response to ICIs, which can have implications for the personalization of cancer immunotherapy.

The CDKN2A/2B ALTs may result in abnormal activation of CDK2/4/6, so CDK inhibitors serve as a potential treatment for patients with CDKN2A/2B ALTs [47]. The growth of tumor cells is inhibited both in vivo and in vitro when p16INK4a gene is transferred into lung cancer cells, glioma cells and gastric cancer cells without p16INK4a expression by transgenic technology, suggesting that the expressed product has tumor therapeutic effect [48–50]. Clinical study also shows that CDKN2A methylation, mutation, or loss of p16 (INK) protein are associated with increased melanoma susceptibility to CDK4/6 inhibitor Palbociclib [51]. However, Palbociclib has no efficacy in pancreatic and biliary cancer with CDKN2A ALT [47]. Likewise, in another study, patients with p16/CDKN2A deficient imatinib/sunitinib-resistant gastrointestinal stromal tumor have no significant clinical benefits of Palbociclib monotherapy [52]. Intriguingly, elevated tumor cell antigen presentation combined with anti-tumor T cell responses shows that CDK4/6 inhibitors may increase response to ICIs for tumors [53]. In agreement with the above study, a significant correlation of CDKN2A ALT frequency with ORRs was found in the MSK-MetTropism and MSK-IMPACT cohorts. Our study suggests that combined therapy with ICIs and CDK4/6 inhibitors may have additional advantages over their corresponding monotherapies. Currently, there is a clinical trial of patients with ER^+^ breast cancer treated with a combination of three FDA-approved CDK4/6 inhibitors and anti-PD-1/PD-L1 inhibitors (NCT03294694, NCT02778685 and NCT03147287) [54].

In a small clinical study of 20 patients with NSCLC treated with PD-1 blockade, hyperprogressive disease was found five patients, four of whom carried CDKN2A/B ALTs [55]. This result suggests that CDNK2A/B inactivation or copy number loss may be associated with poor immunotherapy response in tumor patients. Next, the positive correlation of PD1/PD-L1 inhibitors with hyperprogressive disease was found in advanced NSCLC and metastatic head and neck squamous cell carcinoma [56, 57]. However, in the MSK-IMPACT cohort of our study the PD1/PD-L1 inhibitors seem to improve the clinical outcome of CDKN2A-MUT patients with NSCLC or head and neck cancer, treated by ICIs, in despite of CDKN2A ALT being found to be a risk factor of survival in the MSK-MetTropism and TCGA cohorts. Encouragingly, 215 CDKN2A-MUT patients with bladder cancer after ICIs treatment had better OS than CDKN2A-WT patients in this study. On the contrary, CDKN2A ALT was associated with poor response to ICIs and OS in 44 urothelial carcinoma patients treated with ICIs from the dana-farber cancer institute [7]. These apparently conflicting results could be due to disease heterogeneity and relatively small sample sizes of 44 urothelial carcinoma patients.

In the MSK-MetTropism cohort, CDKN2A ALT had a worse prognosis in most primary cancer types than metastatic. However, it was noteworthy that CDKN2A ALT was favored in the prognosis of patients treated by ICIs from cancer patients having metastatic disease and not primary cancers patients in the MSK-IMPACT cohort. Similarly, CDKN2A DEL increased the risk of distant metastasis in multiple cancers, including HNSC, KIRC, PAAD, skin cutaneous melanoma (SKCM), and stomach adenocarcinoma (STAD) in TCGA cohort [58]. Specifically, CDKN2A ALT reduces cancer cell apoptosis and increases the migration and invasion of cancer cells by downregulating P53 expression and upregulating RB1 phosphorylation [59]. Theoretically, the increase of metastatic tumor sites and neoantigens caused by CDKN2A ALT provides an opportunity for the tumor-infiltrating white blood cells (TILs) to recognize and attack cancer cells in the tumor tissues. Of importance, compared to primary tumor sites with more immune cell depletion, new metastatic sites might have more abundant tumor immune cells to improve the therapeutic efficiency of ICIs.

One notable finding was that in the MSK-IMPACT cohort, patients with CDKN2A MUT exhibited a notably improved OS following treatment with ICIs compared to patients with CDKN2A WT. Conversely, in TCGA dataset, we assessed the extent of immune cell infiltration using CIBERSOFT and observed that CDKN2A-MUT, CDKN2A-ALT, or CDKN2A-DEL cases displayed reduced immune cell infiltration and lower expression levels of crucial genes associated with tumor immune function. Moreover, earlier investigations have demonstrated that CDKN2A DEL is associated with the establishment of a cool tumor immunological microenvironment [12, 60, 61]. ‘Cold tumors’ is a term used to describe tumors that have a limited or weak immune response [62]. These tumors typically have a low level of immune cell infiltration and lack the presence of certain immune-stimulating molecules [63]. As a result, they are less likely to respond to immunotherapy, which aims to stimulate the immune system’s ability to recognize and destroy cancer cells [64]. Research suggests that certain factors, such as CDKN2A ALT, confers a cold tumor immune microenvironment and influence the immune response to immunotherapy [12]. Interestingly, our findings also revealed that CDKN2A-ALT patients displayed a cold tumor immune microenvironment in gastric cancer single-cell data analysis. However, it is noteworthy that the OS of CDKN2A-ALT patients who received ICIs in the MSK-IMPACT cohort was remarkably superior to that of CDKN2A WT patients. This paradoxical result can potentially be explained by several factors.

First, in our study, patients with CDKN2A ALT had more elevated TMB than those with CDKN2A WT. Elevated TMB can result in the production of more tumor-specific antigens (TSAs), which can enhance the recognition and targeting of tumors by the immune system. This increased antigenicity may contribute to better responses to ICIs, even if there is less immune cell infiltration in the tumor microenvironment [65]. The recognition and attack of these antigens by ICIs can potentially inhibit tumor growth and improve treatment outcomes. This highlights the complex interplay between TMB, antigen presentation, and the efficacy of immunotherapy in patients with CDKN2A ALT.

Second, within the TCGA cohort, it was observed that patients with CDKN2A-ALT exhibited decreased expression levels of immunosuppressive genes, including CTLA4 and IDO1. Furthermore, an examination of single-cell data in gastric cancer has demonstrated that tumors originating from patients with CDKN2A-ALT exhibit a higher abundance of fatigued CD8 T cells and a lower abundance of regulatory T cells compared to tumors from CDKN2A WT individuals. The activation of latent anti-tumor T cell responses with immune treatment has the potential to reawaken immune memory against the tumor [66]. In the context of “cold” tumors, it is noteworthy that a limited population of anti-tumor immune cells exists, which holds the potential to exert a favourable influence on therapeutic interventions [67].

Ultimately, clinical investigations have provided data indicating that a deficiency in the quantity of immune infiltrating cells within the tumor could potentially enhance the efficacy of immunotherapy [68, 69]. Insufficient infiltration of immune cells may lead to the immune system’s failure in recognizing cancer antigens [70]. Immunotherapy enhances the immunological response of a patient’s immune system in identifying cancer antigens, hence augmenting the overall efficacy of the therapy in combating the disease. The efficacy of immunotherapy against tumors may be attributed, in part, to the lack of immune cell infiltration within the tumor [71]. In brief, it is worth noting that this is an area of ongoing research, and the specific mechanisms underlying the sensitivity of CDKN2A-altered tumors to immunotherapy are still being explored. Further investigations and clinical studies are needed to fully understand and develop this concept.

### Limitations

Our study has several limitations. First, the second validation cohort comprised 10 194 patients from the Chinese population and did not have clinical outcomes to assess the association between CDKN2A ALT frequency and prognosis. Second, although CDKN2A ALT was inversely associated with the OS of patients treated without ICIs and positively associated with the OS of patients, especially patients having metastatic disease, treated with ICIs, the relationship and underlying mechanisms behind this association need to be further assessed. Third, the lack of CDK4/6 inhibitors treatment limited the analysis of interaction effects between ICIs and CDK4/6 inhibitors on clinical outcomes of pan-cancer patients with CDKN2A ALT. Fourth, only single-cell data from CDKN2A point mutation study of stomach cancer were given, not those from single-cell analysis of CDKNA DEL patients. Therefore, further in-depth examination of combined therapy with ICIs and CDK4/6 inhibitors in pan-cancer patients with CDKN2A ALT will be necessary in future studies.

## Conclusions

To our knowledge, this cohort study with the largest sample size determines the association of CDKN2A ALT with clinical outcomes and response to ICIs among pan-cancer patients treated or not treated with immunotherapy to provide a reference for better personalizing therapy for CDKN2A ALT cancer patients. Overall, CDKN2A ALT remain an important area of research in cancer immunotherapy, and further studies are needed to clarify the role of CDKN2A mutations/deletions as potential biomarkers for ICI treatment.

### Electronic supplementary material

Below is the link to the electronic supplementary material.

Supplementary Material 1

Supplementary Material 2

Supplementary Material 3

Supplementary Material 4

Supplementary Material 5

Supplementary Material 6

Supplementary Material 7

Supplementary Material 8

## Data Availability

The public datasets were obtained from cBioPortal (https://www.cbioportal.org) and UCSC Xena data portal (https://xenabrowser.net). All other data will be made available on request.

## Abbreviations

CDKN2A/MTS1: Cyclin dependent kinase inhibitor 2 A/multiple tumor suppressor 1
TCGA: The Cancer Genome Atlas
CNV: Copy number variation
ICIs: Immune checkpoint inhibitors
GSEA: Gene set enrichment analysis
TMB: Tumor mutation burden
MSI: Microsatellite instability
TIL: Infiltrating lymphocyte
TP53: CDKN2A, RB1 and BRCA1
CDK4: Cyclin-dependent kinase 4
CDK6: Cyclin-dependent kinase6
BRN2: POU class 3 homeobox 2
BAX: BCL2 associated X, apoptosis regulator
TP53INP1: Tumor protein p53 inducible nuclear protein 1
TP53BP1: Tumor protein p53 binding protein 1
CDKN1A: Cyclin dependent kinase inhibitor 1 A
MDM2: MDM2 proto-oncogene
CDKN1B: Cyclin dependent kinase inhibitor 1B
CCDN1: Cyclin D1
GSEA: Genome set enrichment analysis
UCSC Xena: Xena Functional Genomics Explorer
OS: Overall survival
DSS: Disease-specific survival
DFI: Disease-free interval
PFI: Progression-free interval
KEGG: Kyoto Encyclopedia of Genes and Genomes
GO: Gene Ontology
FDR: False discovery rate
ACC: Adrenocortical carcinoma
BLCA: Bladder Urothelial Carcinoma
BRCA: Breast invasive carcinoma
CESC: Cervical squamous cell carcinoma and endocervical adenocarcinoma
CHOL: Cholangiocarcinoma
COAD: Colon adenocarcinoma
CDKN2A: Is a biomarker for multiple cancers
DLBC: Lymphoid Neoplasm Diffuse Large B cell Lymphoma
ESCA: Esophageal carcinoma
GBM: Glioblastoma multiforme
HNSC: Head and Neck squamous cell carcinoma
KICH: Kidney Chromophobe
KIRC: Kidney renal clear cell carcinoma
KIRP: Kidney renal papillary cell carcinoma
LAML: Acute Myeloid Leukemia
LGG: Brain Lower Grade Glioma
LIHC: Liver hepatocellular carcinoma
LUAD: Lung adenocarcinoma
LUSC: Lung squamous cell carcinoma
MESO: Mesothelioma
OV: Ovarian serous cystadenocarcinoma
PAAD: Pancreatic adenocarcinoma
PCPG: Pheochromocytoma and Paraganglioma
PRAD: Prostate adenocarcinoma
READ: Rectum adenocarcinoma
SARC: Sarcoma
SKCM: Skin Cutaneous Melanoma
STAD: Stomach adenocarcinoma
TGCT: Testicular Germ Cell Tumors
THCA: Thyroid carcinoma
THYM: Thymoma
UCEC: Uterine Corpus Endometrial Carcinoma
UCS: Uterine Carcinosarcoma
UVM: Uveal Melanoma
IL-4: interleukin 4
IL-10: interleukin 10
