# Supplementary material for: Pan-cancer analysis of CDKN2A alterations identifies a subset of gastric cancer with a cold tumor immune microenvironment
Source: Hum Genomics. 2024 May 31;18:55. doi: 10.1186/s40246-024-00615-7 (PMC11143690; doi:10.1186/s40246-024-00615-7)
Supplement: Supplementary file 1 — Supplementary Material 1 [file 40246_2024_615_MOESM1_ESM.docx]

| **Table S1 Patient characteristics in the discovery cohort** | | | | | |
| --- | --- | --- | --- | --- | --- |
| **Characteristic** | WT, N = 21,085 | DEL, N = 1,934 | MUT, N = 1,396 | Other ALT, N = 88 | *p* |
| **Sample Type** | |  |  |  | <0.001 |
| Metastasis | 7,995 (38%) | 994 (51%) | 595 (43%) | 35 (40%) |  |
| Primary | 13,090 (62%) | 940 (49%) | 801 (57%) | 53 (60%) |  |
| **Tumor Purity** | |  |  |  | <0.001 |
| Median (IQR) | 40 (20, 50) | 50 (30, 60) | 30 (20, 50) | 30 (20, 50) |  |
| **FGA** |  |  |  |  | <0.001 |
| Median (IQR) | 0.13 (0.03, 0.30) | 0.27 (0.17, 0.42) | 0.12 (0.02, 0.27) | 0.16 (0.02, 0.34) | |
| **MSI Score** | |  |  |  | <0.001 |
| Median (IQR) | 0.21 (0.00, 0.84) | 0.59 (0.19, 1.29) | 0.21 (0.00, 0.75) | 0.22 (0.00, 0.98) | |
| **TMB** |  |  |  |  | <0.001 |
| Median (IQR) | 4 (3, 7) | 4 (3, 8) | **8** (5, 16) | 3 (3, 8) |  |
| **Sex** |  |  |  |  | <0.001 |
| Female | 11,432 (54%) | 894 (46%) | 613 (44%) | 45 (51%) |  |
| Male | 9,653 (46%) | 1,040 (54%) | 783 (56%) | 43 (49%) |  |
| **Race** |  |  |  |  |  |
| Asian-far east/Indian subcont | 1,473 (7.0%) | 146 (7.5%) | 69 (4.9%) | 6 (6.8%) |  |
| Black or African American | 1,357 (6.4%) | 116 (6.0%) | 54 (3.9%) | 4 (4.5%) |  |
| Other | 901 (4.3%) | 88 (4.6%) | 55 (3.9%) | 3 (3.4%) |  |
| Unknown | 814 (3.9%) | 70 (3.6%) | 58 (4.2%) | 5 (5.7%) |  |
| White | 16,540 (78%) | 1,514 (78%) | 1,160 (83%) | 70 (80%) |  |
| **Age** |  |  |  |  | <0.001 |
| Mean (SD) | 62 (13) | 65 (12) | 66 (12) | 66 (12) |  |
| **Mutation Count** | |  |  |  | <0.001 |
| Median (IQR) | 5 (3, 8) | 5 (3, 9) | 7 (5, 17) | 4 (3, 8) |  |
| **Cancer Type** | |  |  |  |  |
| Anal Cancer | 80 (0.4%) | 0 (0%) | 3 (0.2%) | 1 (1.1%) |  |
| Appendiceal Cancer | 191 (0.9%) | 1 (<0.1%) | 0 (0%) | 0 (0%) |  |
| Bladder Cancer | 839 (4.0%) | 224 (12%) | 57 (4.1%) | 3 (3.4%) |  |
| Breast Cancer | 2,389 (11%) | 74 (3.8%) | 30 (2.1%) | 4 (4.5%) |  |
| Cervical Cancer | 100 (0.5%) | 1 (<0.1%) | 0 (0%) | 0 (0%) |  |
| Colorectal Cancer | 3,413 (16%) | 42 (2.2%) | 63 (4.5%) | 3 (3.4%) |  |
| Endometrial Cancer | 1,264 (6.0%) | 16 (0.8%) | 23 (1.6%) | 0 (0%) |  |
| Esophagogastric Cancer | 638 (3.0%) | 66 (3.4%) | 101 (7.2%) | 4 (4.5%) |  |
| Gastrointestinal Neuroendocrine Tumor | 108 (0.5%) | 8 (0.4%) | 1 (<0.1%) | 0 (0%) |  |
| Gastrointestinal Stromal Tumor | 291 (1.4%) | 92 (4.8%) | 1 (<0.1%) | 1 (1.1%) |  |
| Germ Cell Tumor | 220 (1.0%) | 0 (0%) | 1 (<0.1%) | 0 (0%) |  |
| Head and Neck Cancer | 270 (1.3%) | 44 (2.3%) | 75 (5.4%) | 3 (3.4%) |  |
| Hepatobiliary Cancer | 724 (3.4%) | 97 (5.0%) | 40 (2.9%) | 6 (6.8%) |  |
| Melanoma | 686 (3.3%) | 262 (14%) | 171 (12%) | 4 (4.5%) |  |
| Mesothelioma | 135 (0.6%) | 74 (3.8%) | 4 (0.3%) | 2 (2.3%) |  |
| **Non-Small Cell Lung Cancer** | **3,681 (17%)** | **491 (25%)** | **329 (24%)** | **31 (35%)** |  |
| Ovarian Cancer | 1,096 (5.2%) | 35 (1.8%) | 6 (0.4%) | 4 (4.5%) |  |
| Pancreatic Cancer | 1,247 (5.9%) | 270 (14%) | 398 (29%) | 14 (16%) |  |
| Prostate Cancer | 1,881 (8.9%) | 35 (1.8%) | 11 (0.8%) | 4 (4.5%) |  |
| Renal Cell Carcinoma | 398 (1.9%) | 9 (0.5%) | 3 (0.2%) | 0 (0%) |  |
| Salivary Gland Cancer | 127 (0.6%) | 6 (0.3%) | 1 (<0.1%) | 0 (0%) |  |
| Skin Cancer, Non-Melanoma | 48 (0.2%) | 5 (0.3%) | 49 (3.5%) | 0 (0%) |  |
| Small Bowel Cancer | 81 (0.4%) | 6 (0.3%) | 6 (0.4%) | 0 (0%) |  |
| Small Cell Lung Cancer | 297 (1.4%) | 5 (0.3%) | 6 (0.4%) | 4 (4.5%) |  |
| Soft Tissue Sarcoma | 366 (1.7%) | 38 (2.0%) | 7 (0.5%) | 0 (0%) |  |
| Thyroid Cancer | 381 (1.8%) | 22 (1.1%) | 8 (0.6%) | 0 (0%) |  |
| Uterine Sarcoma | 134 (0.6%) | 11 (0.6%) | 2 (0.1%) | 0 (0%) |  |
| Unless indicated otherwise, data are expressed as No. (%) of patients. Percentages have been rounded and may not total 100.  Abbreviations: NA, not applicable; CNA, copy number alternation; MSI, microsatellite instability; TMB, tumor mutation burden.  ^2^Pearson's Chi-squared test; Kruskal-Wallis rank sum test | | | | | |
