# Supplementary material for: Pan-cancer analysis of CDKN2A alterations identifies a subset of gastric cancer with a cold tumor immune microenvironment
Source: Hum Genomics. 2024 May 31;18:55. doi: 10.1186/s40246-024-00615-7 (PMC11143690; doi:10.1186/s40246-024-00615-7)
Supplement: Supplementary file 2 — Supplementary Material 2 [file 40246_2024_615_MOESM2_ESM.docx]

**S Figure legend**

**Figure S1**. Forest plot of association between CDKN2A ALT and OS of patients diagnosed with metastatic cancer **(A)** in the MSK-MetTropism cohort. Forest plot of association between CDKN2A MUT and OS of all patient (**B**), and patients diagnosed with primary cancer (**C**) and patients diagnosed with metastatic cancer **(D)** in the MSK-MetTropism cohort. Forest plot of association between CDKN2A DEL and OS of all patient (**E**), and patients diagnosed with primary cancer (**F**) and patients diagnosed with metastatic cancer **(G)** in the MSK-MetTropism cohort. **(H)** The association between CDKN2A ALT and OS in prespecified subgroups. **(I)** Correlation between CDKN2A ALT frequency and objective response rates to ICIs.

**Figure S2**. (**A**) The association between CDKN2A ALT and OS in 1661 patients from the MSK-IMPACT cohort. (**B**) Forest plot of association between CDKN2A-ALT and OS. **(C)** OS of CDKN2A-DEL, CDKN2A-MUT, CDKN2A-WT and CDKN2A-other ALT groups with combination treatment.

**Figure S3**. (**A**) The distribution of CDKN2A-DEL, CDKN2A-MUT and CDKN2A-WT in the TCGA cohort. Forest plot of association between CDKN2A MUT(**B**) and DEL(**C**) and PFS. (**D**) Correlation between CDKN2A ALT frequency and objective response rates to ICIs. (E) The association between CDKN2A ALT and OS in the merged cohort of the MSK-MetTropism cohort and TCGA cohort. (F) Forest plot of association between CDKN2A ALT and OS in the merged cohort.

**Figure S4. (A)** Distribution of CDKN2A alterations subgroups in the OrigiMed cohort. The relationship of CDKN2A alterations subgroups with age **(B)**, gender **(C)**, tumor stage **(D)** and therapy ways **(E).**

**Figure S5.** Associations of CDKN2A MUT (**A**) and DEL(**B**) with immune-related genes in TCGA cohort.

**Figure S6.** (**A**) tSNE plot color-coded for 15 clusters of all single cells. (**B**) Dot plot of marker genes of CD8+ T cell clusters.
