# Supplementary figures and images for: Pan-cancer analysis of CDKN2A alterations identifies a subset of gastric cancer with a cold tumor immune microenvironment
Source: Hum Genomics. 2024 May 31;18:55. doi: 10.1186/s40246-024-00615-7 (PMC11143690; doi:10.1186/s40246-024-00615-7)

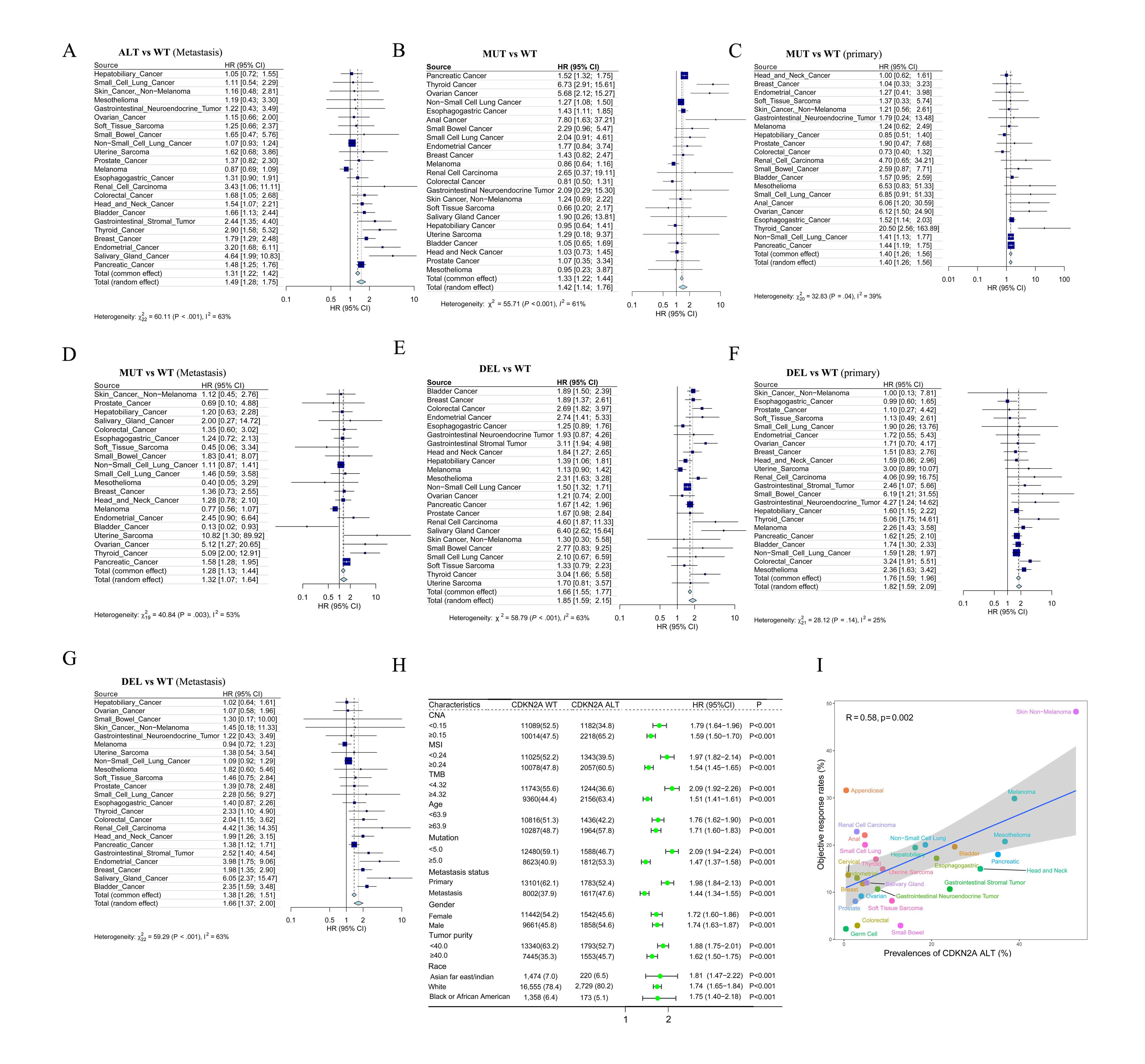

Supplement: Supplementary file 3 — Supplementary Material 3 [file 40246_2024_615_MOESM3_ESM.jpg]

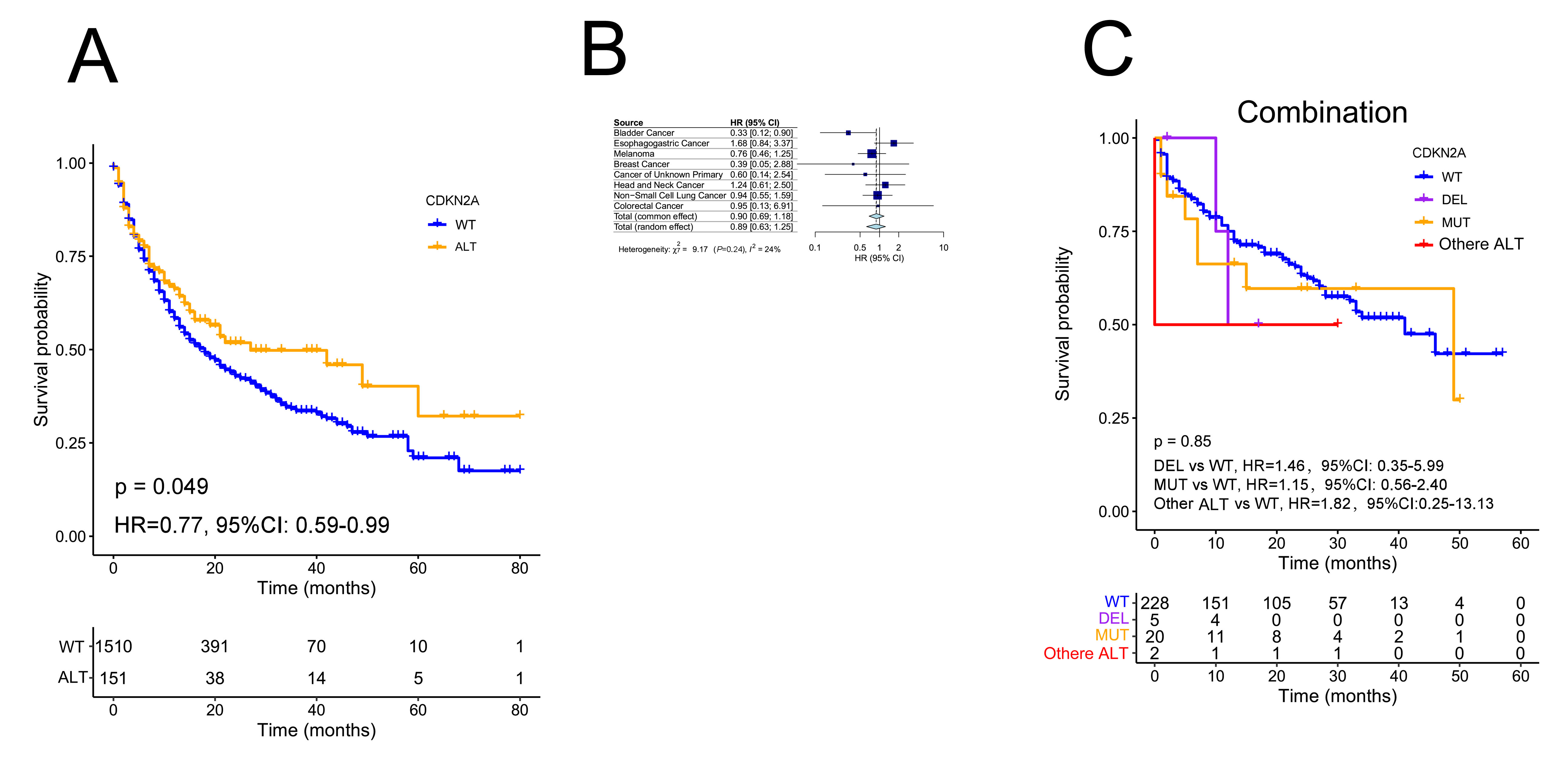

Supplement: Supplementary file 4 — Supplementary Material 4 [file 40246_2024_615_MOESM4_ESM.jpg]

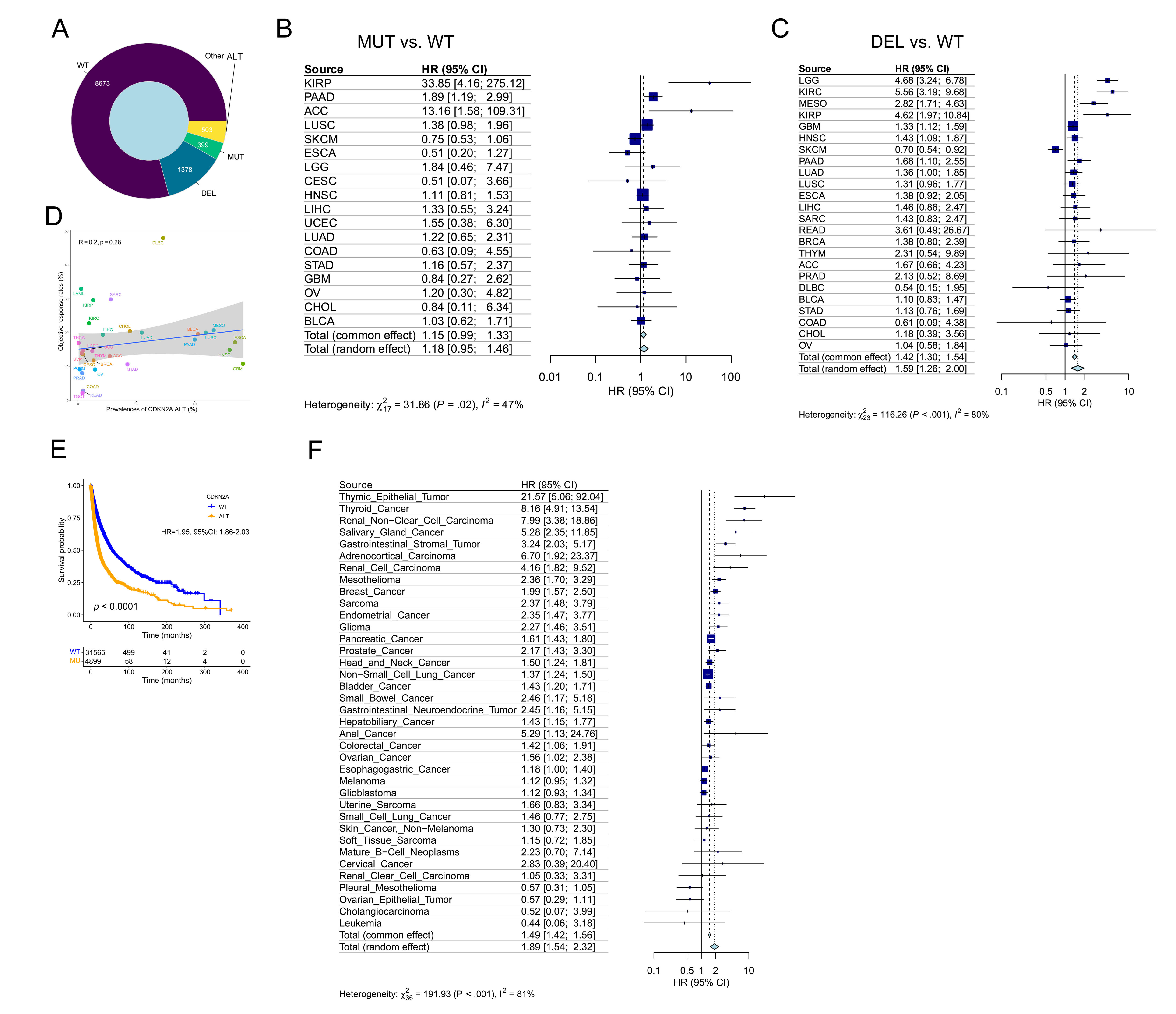

Supplement: Supplementary file 5 — Supplementary Material 5 [file 40246_2024_615_MOESM5_ESM.jpg]

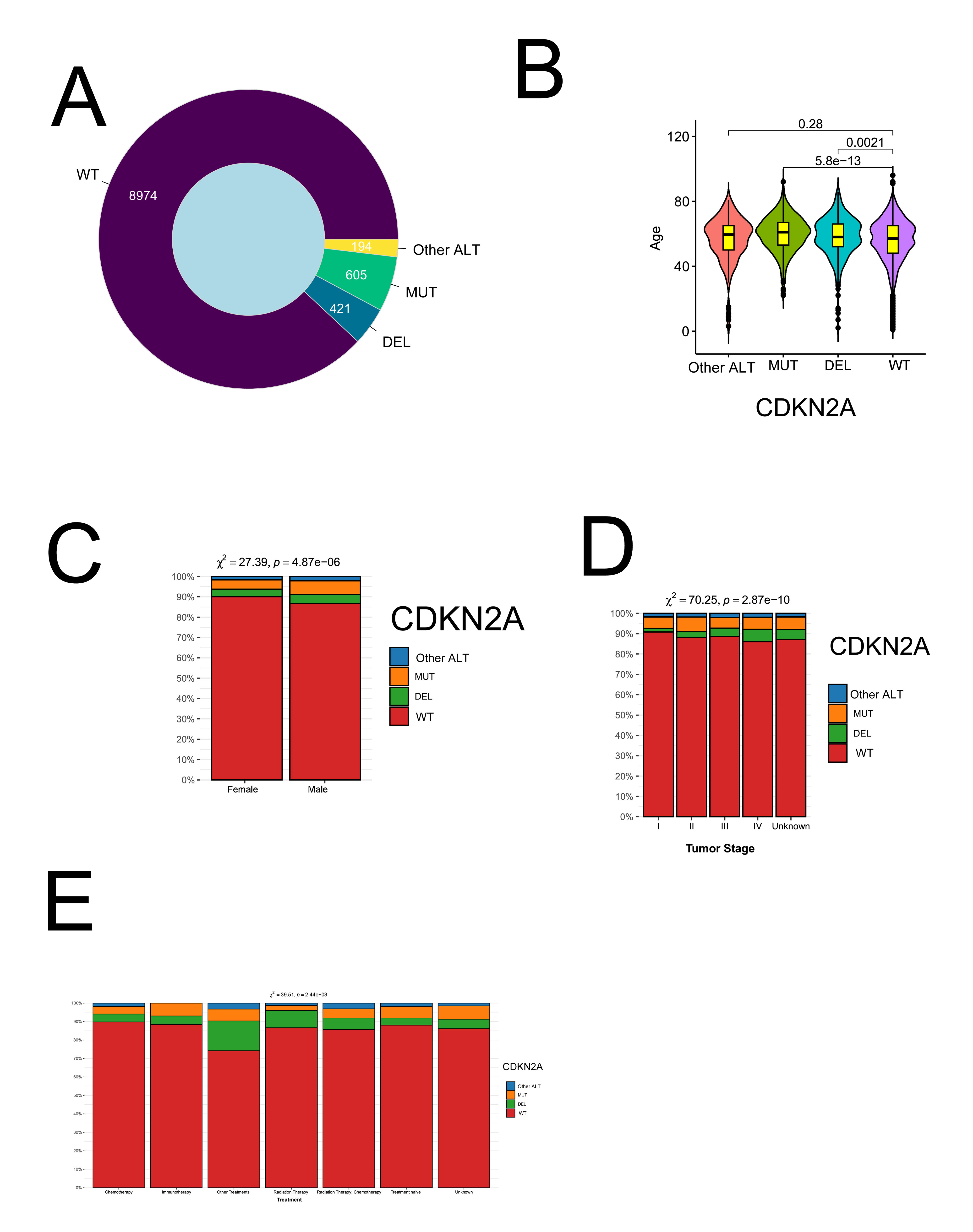

Supplement: Supplementary file 6 — Supplementary Material 6 [file 40246_2024_615_MOESM6_ESM.jpg]

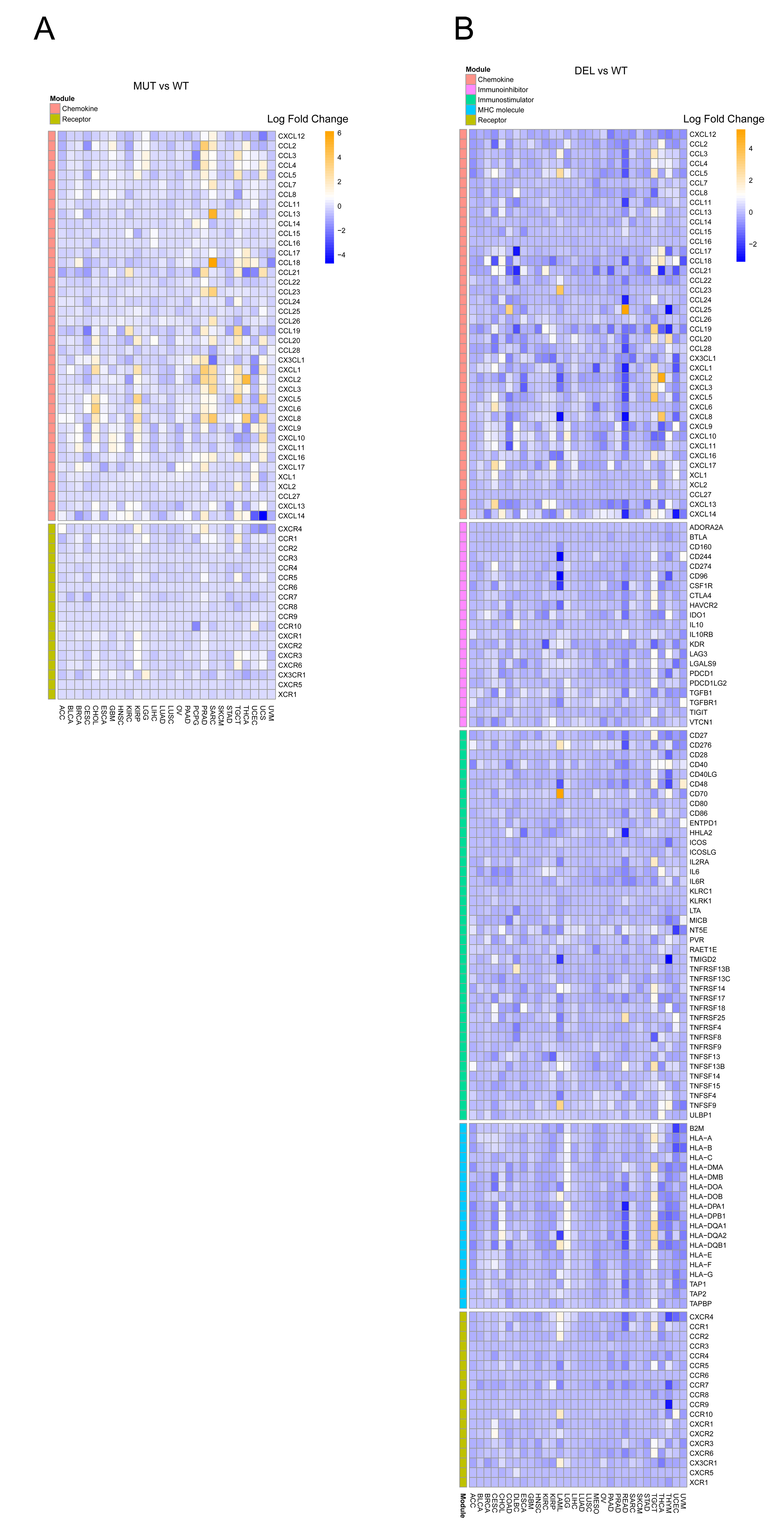

Supplement: Supplementary file 7 — Supplementary Material 7 [file 40246_2024_615_MOESM7_ESM.tif]

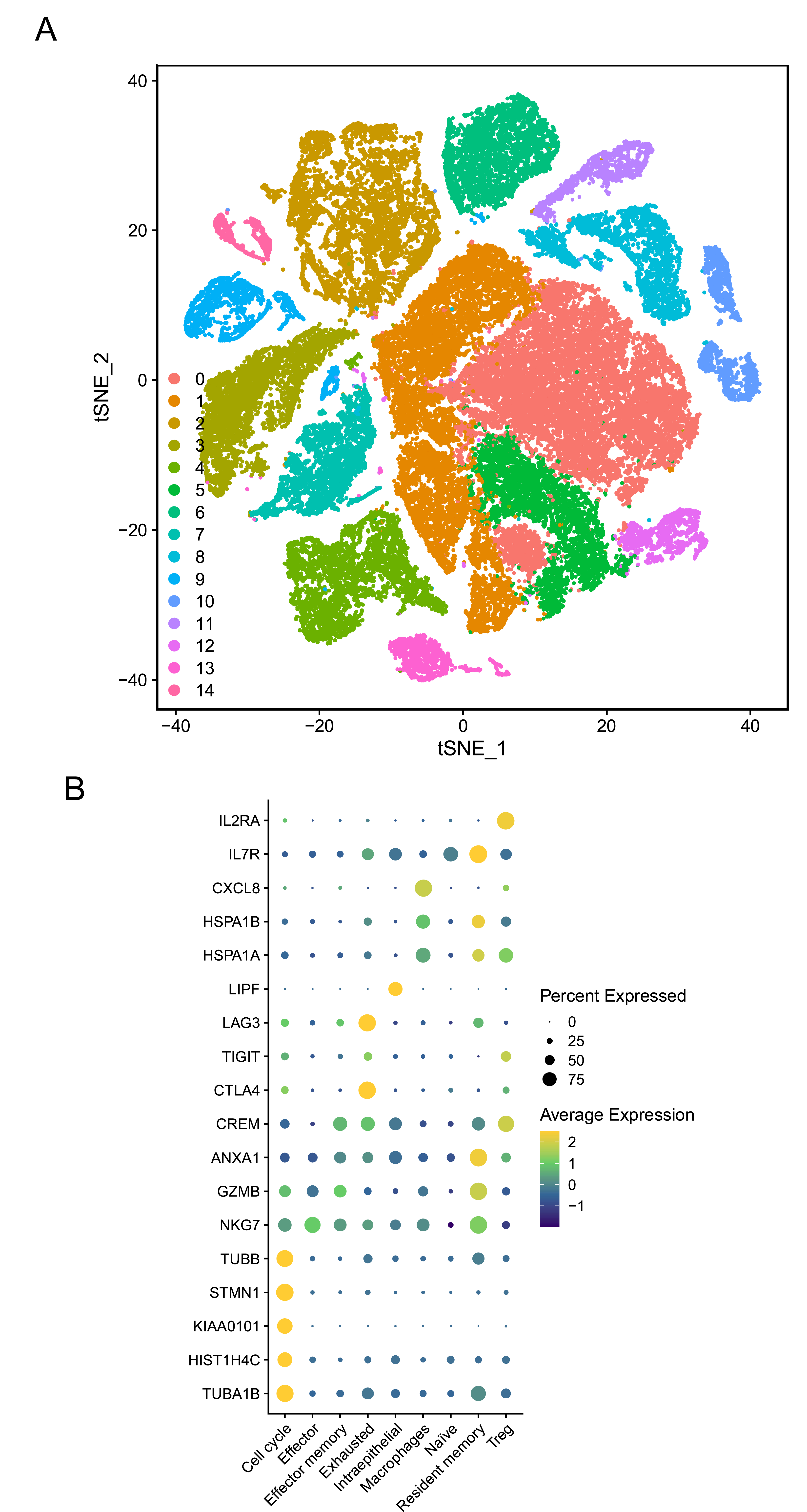

Supplement: Supplementary file 8 — Supplementary Material 8 [file 40246_2024_615_MOESM8_ESM.tif]
